# Depletion of Trypanosome CTR9 Leads to Gene Expression Defects

**DOI:** 10.1371/journal.pone.0034256

**Published:** 2012-04-20

**Authors:** Benard A. Ouna, Benson Nyambega, Theresa Manful, Claudia Helbig, Matilda Males, Abeer Fadda, Christine Clayton

**Affiliations:** Zentrum für Molekulare Biologie der Universität Heidelberg, DKFZ/ZMBH Alliance, Heidelberg, Germany; University of Texas-Houston Medical School, United States of America

## Abstract

The Paf complex of Opisthokonts and plants contains at least five subunits: Paf1, Cdc73, Rtf1, Ctr9, and Leo1. Mutations in, or loss of Paf complex subunits have been shown to cause defects in histone modification, mRNA polyadenylation, and transcription by RNA polymerase I and RNA polymerase II. We here investigated trypanosome CTR9, which is essential for trypanosome survival. The results of tandem affinity purification suggested that trypanosome CTR9 associates with homologues of Leo1 and Cdc73; genes encoding homologues of Rtf1 and Paf1 were not found. RNAi targeting CTR9 resulted in at least ten-fold decreases in 131 essential mRNAs: they included several that are required for gene expression and its control, such as those encoding subunits of RNA polymerases, exoribonucleases that target mRNA, RNA helicases and RNA-binding proteins. Simultaneously, some genes from regions subject to chromatin silencing were derepressed, possibly as a secondary effect of the loss of two proteins that are required for silencing, ISWI and NLP1.

## Introduction

The Paf complex has been implicated in several different aspects of gene expression within the nuclei of yeast and mammalian cells (Opisthokonts) and plants: defects have been shown to influence transcriptional elongation and termination, mRNA processing and export, and to result in epigenetic changes [1]. In *Saccharomyces cerevisiae*, the Paf complex consists of five subunits: Paf1, Cdc73, Rtf1, Ctr9, and Leo1; the human complex also contains Ski8 [1]. The complex is not essential in yeast: but although individual deletions of each subunit are viable, deletions of either *PAF1* or *CTR9* caused decreases in at least 300, and increases in over 900 mRNAs; over half of these were affected in similar ways by both deletions [2]. In contrast, mutation of the human homologues of Cdc73 (HRPT2/CDC73 or parathrombin), loss of Ctr9, and over-expression of Paf1 have all been associated with cancer [1], and a parathrombin missense mutant both impaired nucleolar localization and promoted growth and survival of NIH3T3 cells [3]. In zebrafish, mutation in Rtf1 or Leo1 led to developmental defects [4], [5], while in *Arabidopisis*, the complex is involved in transcriptional control of flowering [6].

The complex has been shown to bind to RNA polymerase II; mutations in either Cdc73 or Rtf1 abolished the binding and resulted in reduced phosphorylation of the C-terminal domain or the largest polymerase II subunit [7]. The complex also stimulates elongation on chromatin substrates, and this effect was not secondary to changes in histone modification [8]. By chromatin IP, the complex was found throughout polymerase-II-transcribed regions of the yeast genome, but association stopped upstream of the polyadenylation site [9]. In both human cells and yeast, an interaction of the Paf1 subunit with a MAP kinase blocked transcription termination on specific genes [10].

Epigenetic changes associated with the Paf complex include histone methylation, ubiquitylation and acetylation. The Rtf1 component is implicated in histone ubiquitylation [11], [12], histone [13] and DNA methylation [14]; but association of Rtf1 with the rest of the complex may not always be required for its activity [13]. Meanwhile the human Cdc73 homologue parathrombin has been shown to be required for very specific types of histone H3 methylation [15]; the complex was also shown to affect histone acetylation *in vitro* [8].

Defects in transcription elongation can affect choices between alternative polyadenylation and splice sites [16], [17], [18], [19]. One possible reason is that there are effects on pre-RNA secondary structure; a second possibility is that if polymerase pauses after synthesis of one, rather inefficient, processing site, there may be time for that site to be used before an alternative, more efficient downstream site has been synthesised. Thus although mutations in the Paf complex can result in changes in mRNA processing, it is not clear how many of the effects are direct. For example, yeast Paf deletion mutants show changes in poly(A) site selection (e.g. [2]); and the complex directly interacts with polyadenylation factors [7], [20]. In addition, Leo1 interacts directly with RNA *in vitro* [21] and is necessary, and sufficient for association of the complex with RNA: thus association of the complex with chromatin might be RNA-mediated [21].

Very few studies have examined possible roles of the Paf1 complex in RNA polymerase I transcription. One found that Paf1 was required for histone K4 methylation on the rDNA [22], which is usually associated with silencing. In contrast, another group found that the Paf1 complex was associated with rDNA, and stimulates polymerase I elongation [23]: deletions in Cdc73, Ctr9 or Paf1 resulted in polymerase I transcription defects [24].

African trypanosomes multiply in the blood and tissue fluids of mammals (as the “bloodstream form”) and in Tsetse flies, where the form found in the midgut is called the “procyclic form”. Trypanosomes offer an interesting model for the study of RNA polymerase II transcription. Genes are arranged in polycistronic transcription units, which are transcribed from several start points [25] located in regions that are marked by specific histone modifications [26], but not by any obvious sequence conservation. Once initiation has occurred, elongation can continue for over 150 kb [26]. Termination sites are enriched in specific histone variants and are often followed by tRNA genes [26]. Individual mRNAs are generated by cotranscriptional processing – addition of a capped 39nt leader at the 5′ end by *trans* splicing, and 3′ polyadenylation [27]. The spliced leader genes, which encode the capped leader plus an intron, are the only trypanosome polymerase II transcription units that are monocistronic and have unique start sites. Once mRNAs have been produced, their abundances are regulated by differential degradation [28], [29], [30].

Proteins involved in trypanosome polymerase II transcription apparatus differ in many ways from those of Opisthokonts. The largest subunit of RNA polymerase II lacks the characteristic heptapeptide repeats at the C-terminus, but that region is nevertheless serine-rich, phosphorylated, and required for transcription [31]. Various transcription factors have been identified [32]. Some are identifiable (although often extremely diverged) homologues of known transcription factors from other eukaryotes, but others are not. For example, the spliced leader promoter-binding complex has a Kinetoplastid-specific subunit [33]; ENF, a protein that interacts with TfIIB, has various motifs that are found in transcription factors, but has no clear Opisthokont homologue [34]; and the trypanosome Mediator complex resembles that of yeast in overall shape (as judged by electron microscopy) but not in the protein primary structures [35], [36].

*Trans* splicing enables trypanosomes to produce protein-coding mRNAs using RNA polymerase I. The genes encoding the Variant Surface Glycoprotein (VSG) of the bloodstream forms, together with several associated genes, are transcribed by polymerase I from telomeric “expression sites”, while the EP and GPEET procyclins of the procyclic form, with a few downstream “procyclin associated genes” (*PAG*) are transcribed from chromosome-internal locations [37]. In both cases, the polymerase I transcription is subject to developmental regulation, which suppresses inappropriate expression; this is at lest partially chromatin-mediated [38], but specific factors may also be involved [36]. Transcription of the *EP* and *GPEET* procyclin genes is about ten times more active in procyclic forms than in bloodstream forms [36], [39]; in addition, procyclin RNAs are very rapidly degraded in bloodstream forms and stable in procyclic forms (reviewed in [29]).

In this paper, we investigated the function of CTR9 in trypanosomes. We found evidence for interaction between trypanosome CTR9, CDC73, and a putative homologue of LEO1, and observed multiple effects on the transcriptome after CTR9 depletion.

## Methods

### Trypanosomes and Plasmids

For RNAi, a TbCTR9-specific DNA fragment was cloned into the vector p2T7TA blue [40] using the primers 5′-GGCACCCTGCAGAATATTCT-3′ and 5′-CGGAGGTAAATTTGGCCGAG-3′. The resulting plasmid was linearized with Not I and transfected into bloodstream-form trypanosomes expressing the tet repressor (pHD 1313) and T7 RNA polymerase (pHD514). Transfectants were selected in 10 mg/ml hygromycin and cloned by limiting dilution. For the growth curve shown, RNAi was induced by adding tetracycline to the medium, at a concentration of 1 mg/ml. Samples were taken after every 24 hours of RNAi induction for three days. In later experiments, 100 ng/ml tetracycline was used, with similar effects on cell growth.

For expression of C-terminally myc-tagged CTR9, the gene was amplified by standard PCR using the following oligonucleotides: 5′- GGGCCCATGCAATATATATCGGAACCC-3′ and 5′- GTTAACTATATTCCCATCATTAGCGGTGT-3′. The fragment was cloned into pHD1700 [41] at the *Apa* I and *Hpa* I sites (pHD2244), and expression induced using 100 ng/ml tetracycline. Expression of the C-terminally TAP-tagged version (IgG binding domain and calmodulin binding peptide) was achieved similarly by cloning into pHD 918 to give pHD2245 [42], [43].

### Protein Analysis

To determine the subcellular location of CTR9, approximately 5×10^8^ bloodstream cells expressing TAP or myc-tagged CTR9 were collected by centrifugation and analysed using NP-40 lysis and centrifugation [44]). Proteins were analysed by Western blotting, and detected by enhanced chemiluminescence (Amersham, Braunschweig, Germany) according to the manufacturer’s instructions. TAP- and myc-tagged CTR9 gave similar results.

For tandem affinity purification, expression of TAP-tagged protein was induced for 24 h and about 1×10^10^ cells collected. TAP purification was done as described by [45] and as modified by [42]. Purified proteins were concentrated overnight by TCA, then separated on a 10% uniform SDS-PAGE gel until all the dye had entered the gel. The gel was stained using Colloidal Coomassie. The section containing proteins was sliced into equal pieces and analysed by LC-MS (ZMBH, DKFZ alliance).

### RNA Analysis

RNASeq analysis was done using poly(A)+ mRNA extracted 24 h after tetracycline addition; in the culture used, growth inhibition had not yet become apparent. Methods used were exactly as previously described [30].

RNASeq data for the CTR9 RNAi mutant were compared to those of 2 replicates of poly(A)+ mRNA from wild-type cells [30], using DESeq [46]. Differentially expressed genes are those with a p-adjusted-value <0.05. Comparisons with other datasets were done as follows. Transcription start and stop sites were annotated manually, by scrutiny of histone modifications shown at TritrypDB and published in [26]. The five ORFs that were nearest to start or stop sites were designated as such in the Excel tables, with the proximal ORF annotated as “–1” and the distal one as “–5”. Data for developmental regulation were extracted from [47]. To determine whether genes are essential for growth, we used published RNA interference sequencing data [48]. To determine the fold effect of RNAi in the published screen, the reads at either day 3, or day 6 after tetracycline induction of RNAi [48] were divided by the number of reads at day 0. ORFs showing a decrease of at least 4-fold, coupled with statistical significance (“TRUE” judgement in the Table provided in [48]) were judged to be essential for normal growth. Northern blots were done using standard methods; reverse transcription and PCRs for RNA detection were done as described in [30] using the primers listed in Table S2.

## Results

### Trypanosome CTR9 is an Essential Protein which Co-purifies with Homologues of Leo1 and Cdc73

**Figure 1:**
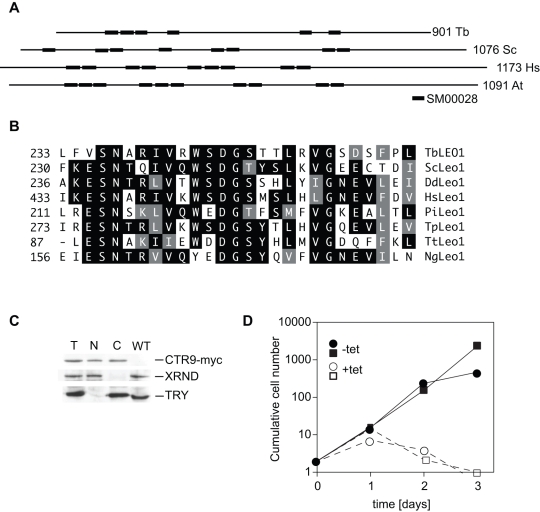
Properties of CTR9 complex components. A. The locations of TPR repeats (SMART SM00028) in trypanosome (Tb), *Saccharomyces cerevisiae* (Sc), human (Hs) and *Arabidopsis thaliana* (At) Ctr9 homologues. The maps are taken from the Interpro scan web site. Domains are shown as thickened bars. B. The most conserved portion of the Leo1 domain. The alignment was made using the MegAlign portion of the DNAStar package; for details see Figure S3. Identical amino acids are underlain in black and functionally conserved amino acids are underlain in grey. Dd: *Dictyostelium disoideum*; Pi: *Phytophthora infestans* – Tp: *Thalassiosira pseudonana*; Tt: *Tetrahymena thermophila*; Ng: *Naegleria gruberi*. C. Myc-tagged CTR9 fractionates into both the nucleus and the cytoplasm. Cells expressing CTR9-myc were lysed with NP40, centrifuged, and the extracts subjected to Western blotting using antibodies to myc (Santa Cruz), XRND (nuclear marker) and trypanothione reductase (cytoplasmic marker). T: total; N: nuclear fraction; C: cytoplasmic fraction. D. Effect of RNAi against CTR9. Two separate bloodstream trypanosome lines (represented by different symbols) were incubated with or without 100 ng/ml tetracycline. The starting cell density was 2×10^5^/ml and cultures were diluted to that level daily as required.

The sequence encoded by Tb927.3.3220 was identified as a Ctr9 homologue by reciprocal BLASTp analysis. Like other Ctr9s, it contains multiple TPR repeats (Figure 1A); identities with human, yeast and plant Ctr9s are 21%, 18% and 19% respectively, with similarities spread throughout the sequence (Figure S1). To identify interaction partners, we expressed a version bearing a C-terminal tandem affinity purification (TAP) tag in bloodstream *T. brucei*. The tagged CTR9 was subjected to two steps of affinity purification and the proteins present in the entire preparation were identified by mass spectrometry. Four potential interaction partners were revealed (Table 1). Most notably, there were potential homologues of Cdc73 (Tb927.11.10230) and Leo1 (Tb927.9.12900). There were also two proteins of unknown function (Tb927.7.4030 and Tb927.3.5070) which have no recognised functional domains and no homologues in non-kinetoplastid eukaryotes; these have not been further investigated. We did not check whether the tagged CTR9 was functional, and tandem affinity purification is a relatively stringent procedure, so additional interaction partners might have been missed.

**Table 1:** Proteins that co-purified with CTR9-TAP.

Identified Proteins	Accession Number	No peptides
**Paf complex subunits**		
CTR9	Tb927.3.3220	48
LEO1	Tb927.9.12900	6
CDC73	Tb927.11.10230	2
**Novel proteins**		
hypothetical protein, conserved	Tb927.7.4030	4
hypothetical protein, conserved	Tb927.3.5070	4
**Possible contaminants**		
calmodulin	Tb927.11.13020	1
60S ribosomal protein L17	Tb927.11.4820	2
microtubule-associated protein 2	Tb10.v4.0052	4
polyubiquitin	Tb927.11.9920	2
hypothetical protein, conserved	Tb11.0840	2
ribosomal protein L3	Tb927.4.1790	2
60S ribosomal protein L12	Tb927.9.14000	4
glyceraldehyde 3-phosphate dehydrogenase, glycosomal	Tb927.6.4280	6
glycerol kinase, glycosomal	Tb927.9.12550	5
retrotransposon hot spot protein	Tb11.0050	2
elongation factor 1-alpha,EF-1-alpha	Tb927.10.2100	6
alpha tubulin	Tb927.1.2340	20
beta tubulin	Tb927.1.2330	23
heat shock protein 70	Tb927.11.11330	3

Proteins were denatured, separated by SDS-PAGE and the whole gel section containing proteins was analysed by mass spectrometry. The numbers of different peptides detected are indicated. Possible contaminants are judged as such because of their appearance in other, unrelated purifications.

Trypanosome CDC73 shows 22%, 21%, and 25% identity with the human, yeast and plant proteins, with more conservation towards the C-terminus (Figure S2). Although only two peptides were detected, this seems likely to be a genuine interaction partner. In contrast, the overall identities for LEO1 are very low: 12%, 17% and 15% respectively; its identification relies exclusively on the presence of a Leo1 domain (E value 1.6 e-5) between positions 217 and 257 (Figure 1B and Figure S3). We searched the trypanosome genome for homologues of the remaining subunits but were unable to find them. To look for an association between CTR9 and RNA polymerase II, we precipitated the CTR9-TAP and examined the precipitate for the presence of the largest subunit: no association was seen (Figure S4).

Extrapolating from other systems, we expected to find CTR9 in the nucleus. We therefore examined the protein by cell fractionation. The control, XRND, was retained in the nucleus while trypanothione reductase was found in the cytoplasmic fraction (Figure 1C). In contrast, the 100 kDa C-terminally myc-tagged CTR9 protein was found in both cytoplasm and nucleus (Figure 1C). In repeat experiments with either myc- or TAP tagged proteins, and using half as much tetracycline, the signals were less strong but the protein was still found in both compartments, with slightly more in the nucleus (not shown). It is however possible that the localization of the tagged protein was influenced by the presence of the tag.

### Conservation of PAF Complex Subunits in Evolution

To investigate the evolutionary conservation of the PAF complex, we searched eleven genomes representing all major eukaryotic branches (except for Rhizaria), using the yeast, human or trypanosome sequences as queries. We used standard NCBI BLASTp settings without filters, and matches with poor coverage were checked by reciprocal BLAST. Where results were negative, we increased the sensitivity by searching individual genomes for Ctr9, Cdc73 and Leo1, and for the Leo1 domain alone (Figure 1B). Results (Table 2) suggest that the pentameric complex could be present in plants, and in some, but not all, unikonts (Opisthokonts and some Amoebozoa). Cdc73, Leo1 and Ctr9 were conserved more widely in eukaryotic evolution, but no sequences resembling any of the subunits were found in either *Trichomonas* or *Giardia*. Evidently poor conservation could have compromised the searches, but it does seem that Cdc73, Ctr9 and Leo1 are more widely distributed than Paf1 and Rtf1.

**Table 2:** Putative homologues of the Paf complex subunits in various organisms.

Group/Kingdom	Species	Ctr9	Cdc73	Leo1	Paf1	Rtf1
Plantae	*Arabidopsis thaliana*	B5X0I6_ARATH B5X0I	At3g22590	At5g61156	At1g79730	At1g61040
Opisthokonts	*Saccharomyces cerevsiae*	NP_014496.2	NP_013522.1	NP_014766.1	NP_009838.1	NP_011270.1
Opisthokonts	*Schizosaccharomyces pombe*	NP_594620.1	NP_595891.1	NP_596263.1	NP_593451.1	NP_595507.1
Opisthokonts	*Homo sapiens*	NP_055448.1|	NP_078805.3	NP_620147.1	NP_061961.2	NP_055953.3
Amoebozoa	*Dictyostelium discoideum*	XP_642310.1	XP_644100.3	XP_637916.1	XP_645948.1	XP_637691.1
Amoebozoa	*Entamoeba histolytica*	XP_650475.1	XP_648719.1			
Alveolata	*Tetrahymena thermophila*	EAR82060.1 (and others)	EAR84002.1	EAR89919.2		
Alveolata	*Plasmodium falciparum*	–	XP_001351765.1	–		
Stramenopila	*Thalassiosira pseudonana*	XP_002287483.1	XP_002287950.1	XP_002289477.1		
Heterolobosea	*Naegleria gruberi*	XP_002679971.1	XP_002683436.1		XP_002670362.1	
Euglenozoa	*Trypanosoma brucei*	Tb927.3.3220	Tb927.11.10230	Tb927.9.12900		

No homologues were found in either *Trichomonas* or *Giardia*. More details are in Table S3.

### Depletion of Ctr9 Results in Increased mRNA from the Procyclin Loci

In a high-throughput RNAi screen *CTR9*, *LEO1* and *CDC73* were all found to be essential for normal growth of both bloodstream and procyclic trypanosomes [48]. We indeed found that RNAi targeting *CTR9* in bloodstream forms resulted in growth arrest after 1 day of tetracycline induction (Figure 1D). To examine the reason for the growth arrest, we analysed the poly(A)+transcriptome of the cells 24 h after tetracycline addition.

**Figure 2:**
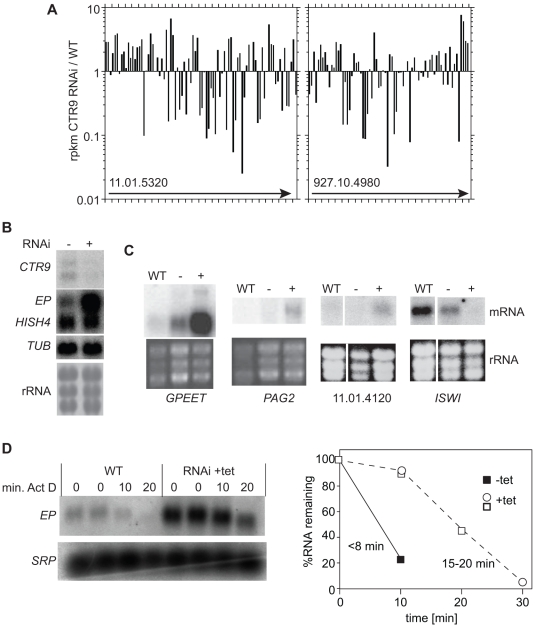
Effects of *CTR9* RNAi on the transcriptome. A. Effect of *CTR9* RNAi on RNAseq representation, for poly(A)+ RNA, across two randomly-selected transcription units starting at loci 11.01.5320 and 927.10.4980. Both units have divergent start and convergent stop sites; in the Figure transcription is from left to right. For the values on the Y-axis, the rpkm for each ORF after 24 h RNAi was divided by the rpkm for wild-type cells [30]. Each bar represents an annotated ORF. B. Northern blot confirmation of the RNASeq results for *EP*, Histone H4 (*HISH4*), and tubulin (*TUB*) ORFs, using new RNA preparations. The rRNA control is from a scan of the methylene blue-stained blot. RNAi was induced for 24 h. C. Northern blot confirmation of the RNASeq results for *GPEET*, *PAG2*, Tb11.01.4120 and *ISWI* ORFs, using new RNA preparations. The rRNA control is a scan of the ethidium-bromide stained gel. D. *CTR9* RNAi effect on *EP* procyclin mRNA abundance and degradation. The left hand panel shows a Northern blot and the graph includes results from two different experiments, one with time points 0, 10 and 20 min and the other with time points 0, 10 and 30 min. Results were normalised to the 7SL RNA from the signal recognition particle (SRP). The half-life of *EP* mRNA is less than 8 min, as previously observed.

Trypanosome polymerase II transcription units contain multiple open reading frames (ORFs). If CTR9 were required for transcription processivity, we would expect that its depletion would result in preferential depletion of mRNAs that are towards the ends of transcription units. This was not seen - increases and decreases were scattered almost randomly through the transcription units (Table S1 and Figure 2A). Table 3 lists the 47 ORFs that show the greatest increases in RNA representation after *CTR9* RNAi. The most dramatic change was an increase in the expression of ORFs from the procyclin transcription units (Table 3). Not only the *EP* and *GPEET* procyclin genes, but also the downstream *PAG*s were affected (Table 3 and Figure 2B). Of the remaining forty ORFs, more than half are located near to rRNA loci, or at transcriptional stop sites (Table 3 and Table S1). This phenomenon was, however, restricted to relatively few chromosomal locations. To find out whether RNA was generally increased near stop sites, we examined 150 ORFs immediately upstream of points where two polymerase II transcription units converge. For these, the median change in transcript abundance was a 2-fold increase: 9 RNAs decreased more than 3-fold, and 48 increased more than 3 fold. Within 10 kb of convergence regions, the effects on individual mRNAs were, however, by no means uniform: one mRNA might go up while its neighbour went down. Figure 2A shows the effect of the RNAi across two transcription units: in one, there is a clear increase at the end, while at the other, there is not. There was no obvious difference in organization between termination points that showed increases and those that didn’t.

**Table 3:** Upregulation of transcript abundance by *CTR9* RNAi.

geneID		rpkm	CTR9/WT	features
**Procyclin locus**				
Tb927.6.520	EP3-2 procyclin	36.4	17	pol I
Tb927.6.510	GPEET2 procyclin	31.3	14	pol I
Tb927.10.10240	PAG1	2.5	48	pol I
Tb927.10.10220	PAG2	1.9	57	pol I
Tb927.6.530	PAG3	12.2	11	pol I
Tb927.10.10210	PAG4	2.6	31	pol I
Tb927.10.10230	PAG5	2.3	37	pol I
**Near rRNA locus**				
Tb09.v2.0450	Unlikely ORF	2.0	17.0	rRNA locus
Tb927.2.1470	hypothetical conserved	3.6	10.0	0 kb, rRNA
Tb927.1.3750	hypothetical conserved	8.7	7.7	0 kb, rRNA
Tb927.3.3430	hypothetical conserved	7.4	7.6	0 kb, rRNA
**Other**				
Tb927.5.4020	hypothetical	4.0	13**	3 kb
Tb927.8.6570	hypothetical conserved	3.4	10	0 kb
Tb927.8.510	hypothetical	1.1	21*	6 kb
Tb927.10.4390	hypothetical	3.6	7.8	3 kb
Tb927.10.4380	hypothetical	0.8	39*	2 kb, ESAG
Tb927.10.12270	hypothetical conserved	4.1	14*	1 kb
Tb927.10.1760	hypothetical	1.7	20*	0 kb
Tb927.11.9380	hypothetical	1.1	14*	0 kb
Tb927.1.2020	hypothetical	3.3	11	0 kb
Tb927.11.11990	hypothetical	1.3	11	0 kb
Tb927.8.480	phosphatase	26.6	9.8	0 kb
Tb927.2.3330	hypothetical	5.3	8.7	0 kb
Tb927.7.310	hypothetical conserved	8.9	8.1	0 kb
Tb927.10.9550	hypothetical	0.6	8.4	6 kb
Tb927.8.5930	hypothetical	0.3	9.4	stop/start
Tb927.3.650	hypothetical conserved	25.8	8.1	11 kb
Tb927.7.2430	Long poly(AT) tracts	0.2	16.9*	repetitive
Tb927.11.4600	hypothetical	1.0	18.5	None
Tb927.10.10040	hypothetical	4.1	13.3	None
Tb927.3.2880	hypothetical conserved	7.6	11.7	None
Tb927.10.9030	hypothetical conserved	10.7	9.7	None
Tb927.3.3570	hypothetical conserved	10.8	9.1	None
Tb927.11.7260	hypothetical conserved	7.8	8.7	None
Tb927.6.4790	hypothetical conserved	33.9	8.2	None
Tb11.v4.0003	hypothetical	2.3	8.2	None
Tb07.26A24.210	hypothetical	4.7	7.8	None
Tb927.3.2370	hypothetical conserved	36.0	7.8	None
Tb927.10.260	hypothetical conserved	29.4	7.7	None
Tb927.10.7720	hypothetical conserved	35.7	7.6	None
Tb927.11.12330	hypothetical conserved	24.5	7.5	None
Tb927.2.2070	hypothetical	3.5	8.6	
Tb927.6.4570	hypothetical conserved	13.8	8.6	start

One representative of each of the procyclin locus ORFs is shown, along with the 36 other ORFs showing the greatest increases in RNA after *CTR9* RNAi. “rpkm” is the reads per kilobase per million reads seen in poly(A)+RNA from normal (wild-type) cells, taken from [30]; the median for all ORFs is 30.6. “CTR9/WT” shows the fold change in rpkm after RNAi (2 significant figures); **no signal was seen on a Northern blot using an ORF probe; *no RNA detected by reverse transcription and PCR targeting the ORF. “Features” indicates transcription by polymerase I (pol I), and the distance of the 3′ end of the locus mRNA from a convergent transcription stop area – “0 kb” indicates that this is the last ORF in a transcription unit. “0 kb, rRNA” indicats that the ORF is the last on a pol II transcription unit, next to an rRNA locus read in the opposite direction. “2 kb, ESAG” is a gene upstream of an ESAG. An rpkm of 20 corresponds to 1 mRNA per cell, which is difficult to detect by Northern blotting.

We also looked to see whether CTR9 depletion changed RNA processing patterns. Predominant splice acceptor sites were mapped as before [30] for 4135 genes with at least 2 reads per site for the *CTR9* RNAi strain. About 37% of these sites were found in the database of predominant sites used that were determined previously on the basis of a collection of WT RNASeq data [30]. Since a similar ratio was obtained when only one wild-type sample was compared to the same database, we concluded that *CTR9* knockdown does not significantly affect the use of splice acceptor sites.

To verify the RNASeq results, we probed Northern blots. The increases in *EP* (Figure 2B), *GPEET* and *PAG2* mRNAs (Figure 2C) were confirmed, as was an increase from locus Tb11.01.4120, an ORF that is not near the end of a transcription unit and showed a 5-fold increase in expression by RNASeq (Figure 2C). Attempts to confirm the up-regulation of some other mRNAs from polymerase II transcription units were however unsuccessful, perhaps due to low abundance (asterisks, Table 3). Some *CTR9*-RNAi-specific reads concentrated in segments of non-coding regions or covered only parts of ORFs; this has not been investigated further. Probing of a Northern blot with a spliced leader probe, to detect all mature mRNAs, did not reveal any global changes in mRNA abundance (not shown).

The increase in *EP* and *GPEET* procyclin mRNAs seemed most likely to be a transcriptional effect, since according to the RNASeq results, the entire transcription units including the *PAGs* were affected: effects on RNA processing or degradation would be unlikely to affect each gene to such similar extents. To check this, however, we assessed the degradation rate of the *EP* procyclin mRNA. Results are shown in Figure 2D. In wild type cells the mRNA has a half-life of less than10 minutes [49]. After *CTR9* RNAi, the onset of *EP* mRNA degradation was reproducibly delayed (Figure 2D), but the apparent 2-3-fold increase in half-life was not, by itself, sufficient to explain the 9-fold increase in mRNA. We therefore concluded that *CTR9* RNAi probably causes a failure of stage-specific transcriptional suppression at the procyclin loci.

In trypanosomes, polyadenylation and *trans* splicing are coupled [50], [51]. To find effects of *CTR9* depletion on RNA processing, we therefore looked for changes in the use of splice acceptor sites, but no significant difference was found (see Methods).

### Depletion of Ctr9 Reduces Expression of Many Genes that are Essential for Control of Gene Expression

We next examined mRNAs that were reduced after *CTR9* RNAi. 349 were decreased at least ten-fold (Table S1). There was no correlation between the effect of *CTR9* depletion and either developmental regulation or half-life (not shown). Even a quick glance, however, was sufficient to explain why *CTR9* RNAi was lethal: no fewer than 131 of the reduced mRNAs are essential for normal growth. Some examples of essential genes with at least 20-fold RNA decreases are shown in Table 4. Multiple genes that encode proteins involved in gene expression now had less than one copy of mRNA per cell: the largest RNA polymerase II subunit and the second largest polymerase I subunit; the TFIIB-interacting protein ENF [34]; two components of the mRNA degradation machinery, NOT1 and XRNA; poly(A) binding protein 2 and some translation factors. There were also other targets potentially involved in gene expression: 7 RNA helicases, including DHH1; zinc finger proteins ZC3H22, ZC3H28, ZC3H32, and ZC3H41, RNA-binding domain proteins DRBD12 and DRBD17, and nearly all of the pumilio domain proteins. mRNAs encoding at least 40 components of the cytoskeleton were reduced, as were four involved in vesicular transport and eleven involved in DNA replication and chromosome maintenance. Correspondingly, an analysis for GO term enrichment showed highly significant over-representation (P values of less than 10^–10^) of gene products in many categories related to nucleotide or nucleoside binding or hydrolysis, as well as motor activity; cytoskeletal categories were enriched with P values of less than 10^–5^ (not shown). Scrutiny of the list also suggested an explanation for deregulation of the procyclin loci: the mRNAs encoding ISWI (Tb927.2.1810) and NLP1 (Tb927.10.5450), were repressed 8-fold and 13-fold, respectively (Table S1 and Figure 2B). RNAi targeting either of these genes deregulates silent expression sites, the procyclin promoters and other silent regions [52], [53].

**Table 4:** Essential genes whose expression is repressed by CTR9 depletion.

geneID	gene description	rpkm	RNAi	BS3		BS6
**Cytoskeleton**						
Tb927.10.14470	IFT140	47.9	0.03	0.02	0.00	
Tb927.10.2640	IFT81	114	0.04	0.00	0.00	
Tb927.10.1170	IFT172	63.8	0.01	0.13	0.23	
Tb927.5.3030	IFT122B	27.7	0.04	0.06	0.25	
Tb927.6.3150	Hydin,flagellar component	19.2	0.04	0.05	0.01	
Tb927.3.5310	paraflagellar rod protein	72.6	0.04	0.10	0.08	
Tb927.11.15100	Paraflagellar rod Tb5.20	142	0.03	0.00	0.00	
Tb927.11.3290	p166	23.9	0.04	0.45*	0.17	
Tb927.4.870	dynein heavy chain	19.3	0.02	0.01	0.01	
Tb927.11.3250	dynein heavy chain	64.5	0.01	0.01	0.02	
Tb927.11.11220	dynein heavy chain	16.0	0.04	0.03	0.04	
Tb927.11.8160	dynein heavy chain	24.9	0.02	0.06	0.04	
Tb927.3.930	dynein heavy chain	63.3	0.03	0.03	0.05	
Tb927.2.5270	dynein heavy chain	20.4	0.02	0.07	0.08	
Tb927.11.2430	dynein heavy chain (DHC1b)	36.1	0.03	0.05	0.08	
Tb927.10.5350	dynein heavy chain	26.9	0.03	0.27*	0.13	
Tb927.8.3250	dynein heavy chain	28.1	0.02	0.06	0.18	
Tb927.7.920	dynein heavy chain	27.3	0.04	0.18	0.21	
Tb927.5.2090	kinesin	41.7	0.04	0.16	0.20	
**Gene expression & DNA replication**						
Tb927.10.1510	NOT1	36.0	0.01	0.18	0.08	
Tb927.7.4900	5'-3' exonuclease XRNA	14.8	0.04	0.15	0.17	
Tb927.10.12660	RNA binding protein PUF2	7.0	0.04	0.15	0.00	
Tb927.9.12510	DEAD/H RNA helicase	58.2	0.05	0.14	0.05	
Tb927.3.2600	DEAD/H RNA helicase	53.8	0.04	0.12	0.16	
Tb927.10.10340	SMC2	34.7	0.03	0.66*	0.14	
**Intracellular protein transport and other**						
Tb927.10.6050	clathrin heavy chain (CHC)	135	0.01	0.01	0.00	
Tb927.10.2900	importin beta-1 subunit	38.6	0.03	0.10	0.02	
Tb927.2.4230	NUP-1, nuclear pore	72.6	0.01	0.17	0.07	
Tb927.10.2880	calcium channel protein	35.7	0.02	0.08	0.04	
Tb927.11.1100	Clan CA cysteine peptidase	48.6	0.01	0.07	0.16	
Tb927.7.2170	hypothetical protein	46.1	0.04	0.10	0.00	
Tb927.7.3550	hypothetical protein	91.3	0.01	0.01	0.00	
Tb927.8.4780	hypothetical protein	33.6	0.02	0.01	0.00	
Tb927.8.2820	hypothetical protein	1801	0.01	0.31*	0.00	
Tb927.9.7690	hypothetical protein	9.3	0.04	0.07	0.01	
Tb927.10.870	hypothetical protein	32.4	0.02	0.08	0.02	
Tb927.9.1750	hypothetical protein	11.0	0.02	0.05	0.02	
Tb927.3.2050	hypothetical protein	20.3	0.03	0.92*	0.04	
Tb927.10.15750	hypothetical protein	114	0.01	0.17	0.05	
Tb927.1.4310	hypothetical protein	23.6	0.01	0.04	0.12	
Tb927.9.13440	hypothetical protein	25.9	0.02	0.20	0.18	
Tb927.7.3560	hypothetical protein	29.1	0.03	0.02	0.23*	

Genes were selected by the following criteria: (a) mRNA decreased more than 20-fold by CTR9 RNAi and (b) A significant decrease of at least 5-fold in representation in the RNAi library, using bloodstream forms either 3 or 6 days after tetracycline addition [48]. “rpkm” is the value for wild-type cells from RNASeq: an rpkm value of 20 is equivalent to one mRNA per cell [30]. “RNAi” is the rpkm for CTR9 RNAi divided by that for wild-type. “BS3” indicates the representation of this gene in RITSeq analysis in bloodstream forms, at day 3 after RNAi induction, divided by the representation without tetracycline; *not significant [48]. “BS6” is the same for 6 days of tetracycline.

## Discussion

We have shown that trypanosome CTR9 co-purifies with homologues of Leo1 and Cdc73 through two affinity purification steps. It is therefore possible that trypanosomes possess a “PAF” complex of at least 3 subunits. The other two subunits, Paf1 and Rtf1, appear to be missing, and a limited phylogenetic analysis suggested that they might be restricted mainly to plants and unikonts. There are two possible explanations. Either Paf1 and Rtf1 equivalents are absent in other eukaryotes, or proteins with no evident sequence similarity are able to substitute functionally for Rtf1 and Paf1. In support of the former supposition, yeast can survive with just Ctr9, Leo1 and Cdc73: deletion of either Rtf1 or Leo1 led to few obvious defects, and although deletion of Paf1 had severe effects, these could be partially abrogated by deletion of either Rtf1 or Leo1 [54]. For the alternative, as noted in the Introduction, functional equivalence in the absence of sequence similarity is already known for some proteins involved in trypanosome transcription. We did identify two additional candidate CTR9 binding partners, Tb927.7.4030 and Tb927.3.5070, but have not verified the association. In the published RNAi screen, Tb927.7.4030 was found to be essential in bloodstream and differentiating forms, and depletion of Tb927.3.5070 inhibited growth [48].

In our affinity purification of tagged CTR9, we did not see co-purification of any subunits of RNA polymerase II, or proteins involved in chromatin modification or RNA processing. Such interactions might not have survived the purification procedure, or they could have been impaired by the presence of the tag. However, it is also very possible that at least some of the interactions that have been seen in other systems do not occur in trypanosomes. For example, yeast [54], [55] and human [8] RNA polymerase II has been shown to coimmunoprecipitate with various Paf complex components, and to copurify with the complex, but the association of the human Paf complex with RNA polymerase II depends on the presence of the PAF1 subunit [8]. Trypanosomes lack an obvious Paf1 homologue, and in the human system, no interaction was seen between polymerase II and purified CTR9, CDC73 or LEO1 [8]. Thus probably, no interaction between the trypanosome CTR9/CDC73/LEO1 is to be expected and indeed, none was found. Thus there is no evidence that the trypanosome CTR9/CDC73/LEO1 complex is involved in polymerase II elongation.

Depletion of CTR9 caused many changes in the transcriptome. To find out whether these were simply a consequence of slowed growth, we compared the most up-and down-regulated genes with those previously detected, by microarray, after other growth-inhibitory treatments: inhibition of glucose transport using phloretin [56], and RNAi targeting *RBP10* [57]. Of the 256 ORFs that showed greater than 3-fold decreases after CTR9 RNAi, only 4 decreased after phloretin (and 10 were more than 1.5-fold increased). Similarly, just 19 of theses same 256 ORFs were 1.5-2-fold decreased after RBP10 RNAi while one increased 1.6-fold. For the 866 *CTR9* RNAi up-regulated genes, only 10 (including *EP* and *GPEET* procyclin genes, but importantly, not *PAG*s) were also at least 1.5-fold increased after *RBP10* RNAi, and 8 after phloretin treatment. The *CTR9* RNAi results also do not resemble those seen after a lethal knock-down of *ZC3H11* (D. Droll, ZMBH, unpublished). This suggests that the transcriptome changes after *CTR9* RNAi were not simply caused by growth inhibition.

By studying the time course of transcriptome changes after Paf1 depletion in yeast, Penheiter et al. [2] found that the first 16 RNAs that were decreased included 11 essential genes; half of them were involved in nucleolar structure and the processing of rRNA [2]. It was therefore possible that many of the subsequent transcriptome changes were secondary to rRNA processing defects. In trypanosomes, we did not study the time course of effects, but certainly within 24 h, CTR9 depletion had decreased so many mRNAs that disentangling primary and secondary effects was not possible. In particular, there were changes in the amounts of many mRNAs encoding proteins that are required for mRNA synthesis and control. First, mRNAs encoding components of RNA polymerases I, II and III decreased to considerably less than one copy per cell: this precludes *in vivo* analysis of specific roles of CTR9 in polymerase II elongation. Next, the expression of NOT1 - a core component of the major deadenylation complex - and of XRNA, a 5′-3′ exoribonuclease that is important for degradation of relatively unstable mRNAs, was abolished. These two essential proteins are required for mRNA degradation. Transcripts encoding many RNA-binding proteins were also decreased; knock-on effects could have occurred if proteins that regulate mRNA degradation were lost while mRNA degradation pathways were still active. Finally, the increase that we observed in polymerase-I-transcribed mRNAs was most likely due to a reduction in chromatin silencing, consequent to the disappearance of ISWI and NLP1.

C-terminally tagged CTR9 was found in both the cytoplasm and the nucleus. Although the tag might have influenced the result, the localisation is in accordance with the PSORTII prediction: the localization percentages of neighbours were 22% nuclear and 61% cytoplasmic for trypanosome CTR9. In contrast, yeast and mammalian proteins yielded 90%, and Drosophila 48%, nuclear scores. It is therefore possible that trypanosome CTR9 functions in both compartments, or is regulated by nucleo-cytoplasmic shuttling.

Overall, our results suggest that trypanosome CTR9 may function as a complex with CDC73, LEO1, and perhaps other proteins. The complex components clearly have essential functions, most likely in gene expression in either the cytoplasm, the nucleus, or both, but the precise natures of these functions remain unclear.

## Supporting Information

Figure S1 **Alignment of Ctr9 homologues, made using MegAlign (DNAStar).** Residues that are identical to the majority consensus are highlighted in black, and residues that are within the consensus group (consensus #1) in grey. Groups are f-hydrophobic; p-polar, a-acidic, b-basic. Organisms are: Tb- *T. brucei*, At- *Arabidopsis thaliana*, NP_178674.6; Dd- *Dictyostelium discoideum* XP_642310; Eh- *Entamoeba histolytica* EAL45089; Hs- *Homo sapiens* NP_055448; Ng- *Naegleria gruberi* XP_002679971; Sc- *Saccharomyces cerevisiae* Ctr9; Sp- *Schizosaccharomyces pombe* CAA15833.
(PDF)

Figure S2 **Alignment of Cdc73 homologues, made using MegAlign (DNAStar).** Details as for Figure S1. Organisms are: Tb- *T. brucei*, Sc- *Saccharomyces cerevisiae*; Sp- *Schizosaccharomyces pombe* NP_595891.1; Hs- *Homo sapiens* NP_078805.3; Dd- *Dictyostelium discoideum* XP_644100.3; Ng- *Naegleria gruberi* XP_002683436.1; Pf- *Plasmodium falciparum* XP_001351765.1; Tt- *Tetrahymena thermophila* EAR84002.1; Pi- *Phytophthora infestans* XP_002903756.1; Eh- *Entamoeba histolytica* XP_648719.1; Tv- *Trichomonas vaginalis* XP_001310227.1; Tp- *Thalassosira pseudonana* XP_002287950.1.
(PDF)

Figure S3 **Alignment of Leo1 homologues, made using MegAlign (DNAStar).** Details as for Figure S1. Organisms are: Tb- *T. brucei*, At- *Arabidopsis thaliana*; Dd- *Dictyostelium discoideum* XP_637916.1; Tp- *Thalassiosira pseudonana* XP_002289477.1; Hs-*Homo sapiens* NP_620147.1; Sc- *Saccharomyces cerevisiae* Leo1; Ng- *Naegleria gruberi* XP_002677260.1; Tt- *Tetrahymena thermophila* EAR92336.1; Pi-*Phytophthora infestans* XP_002903832.1
(PDF)

Figure S4 **TbCTR9-RPB1 co-immunoprecipitation**. Method: Bloodstream-form trypanosomes cell lines expressing TAP-tagged TbCTR9 were used (lanes 5–8), with cells expressing TAP-tag alone as control (lanes 1–4). Protein expression was induced for 24 hrs. 1.5×10^8^ cells were collected by centrifugation and washed once with 1×PBS. Cells were lysed in 500 µl breakage buffer as previously described [42], [45]. The concentration of NaCl was then adjusted to 150 mM and cell debris was removed by centrifugation (4°C) at 13000 rpm in the microfuge for 30 minutes. IgG beads were washed as previously described then clarified cell lysates was added prior to incubation for 2 hrs at 4°C in a microfuge tube rotating at 15 rpm. The unbound fraction was collected by centrifugation. The beads were washed 4× with IPP150 buffer [42], [45] and the protein recovered by boiling the beads in 1× Laemmli lysis buffer. Proteins were separated on an 8% SDS-PAGE gel and transferred overnight to nitrocellulose membranes at 30 V. Protein detection by ECL kit (GE Healthcare) was done using (1∶1000) anti-RPB1 (unpublished, a kind gift from Günzl laboratory); (1∶2000) anti- PAP (peroxidase– antiperoxidase complex; for Protein A detection (Sigma)) for TAP. The loading was equivalent to 5×10^6^ cells for lysate (lanes 1,8) and unbound (Lanes 2,7) fractions; For wash 4 (TCA-concentrated, lanes 3 and 6) and the bead-bound fraction (lanes 4 and 5), the whole preparation was loaded. Results: A good signal of the bound CTR9-TAP protein was detected (lane 5) but there was no detectable RBP1 signal (lane 5). Similar results were obtained by immunoprecipiation of CTR9-myc (not shown).
(PDF)

Table S1 **Effects of CTR9 on the transcriptome.** The first Table is all genes, with ESAGs, VSGs and pseudogenes excluded. The second Table (sheet 2) includes only the “unique genes” of Siegel et al; these are single copy genes or, for repeated genes, a single representative of the gene family. Columns are as follows: A. TritrypDB accession number, v5; B. TritrypDB accession number, v5, but with additional zeros to give each gene ID within a chromosome the same number of digits. This enables a simple sort to arrange the genes in the order in which they appear on the chromosome. C. Annotation in TritrypDB; D. RNASeq results for poly(A)+RNA from cells after 1 day of RNAi targeting CTR9. The reads per kilobase per million reads for the ORF, divided by the ORF copy number (see Manful et al., 2011 for how the copy number was calculated). E. RNASeq results for poly(A)+ RNA from normal cells (see Manful et al., 2011). F. RNASeq results (D) divided by (E). G. Half-life for total RNA (taken from Manful et al., 2011). H. Developmental regulation, taken from Siegel et al., 2010. I. Position of the ORF near a start or stop site, as judged by the enrichment of modified histones as described in Siegel et al., 2009, and displayed on the TritrypDB web site. J. Details of the relative position of the ORF near a start or stop site: the number indicates whether the ORF was the first, second, third etc. relative to the transcription break. K. Nature of start/stop - convergent stops, divergent starts, or stops immediately followed by another start. L. Other notes - gene position near telomeres, pol I transcription, etc. M. Neighbouring locus, if transcribed by pol I or pol III. Only strongly-regulated genes are annotated. N. P-value for regulation (see Methods). Centromeres are from Genome Biol. 2007; 8(3): R37.
(XLSX)

Table S2 **Oligonucleotides used to amplify cDNAs, with comments.**
(XLS)

Table S3 **Details of BLAST search results.** Standard settings were used. Culumns C and D: For Arabidopsis thaliana, the single genome was searched. For all other genomes, the search was done using eleven genomes selected from the NCBI whole genomes database (http://www.ncbi.nlm.nih.gov/sutils/genom_table.cgi). The E values are therefore not comparable with those for Arabidopsis since a much larger database was searched. Columns E and F: Individual genes were blasted back against the S. cerevisiae or H. sapiens databases only. E values are given only if the relevant protein sequence was the best match.
(XLSX)
