# Supplementary material for: Depletion of Trypanosome CTR9 Leads to Gene Expression Defects
Source: PLoS One. 2012 Apr 20;7(4):e34256. doi: 10.1371/journal.pone.0034256 (PMC3332058; doi:10.1371/journal.pone.0034256)
Supplement: Figure S1 — Alignment of Ctr9 homologues, made using MegAlign (DNAStar). Residues that are identical to the majority consensus are highlighted in black, and residues that are within the consensus group (consensus #1) in grey. Groups are f-hydrophobic; p-polar, a-acidic, b-basic. Organisms are: Tb- T. brucei, At- Arabidopsis thaliana, NP_178674.6; Dd- Dictyostelium discoideum XP_642310; Eh- Entamoeba histolytica EAL45089; Hs- Homo sapiens NP_055448; Ng- Naegleria gruberi XP_002679971; Sc- Saccharomyces cerevisiae Ctr9; Sp- Schizosaccharomyces pombe CAA15833. (PDF) [file pone.0034256.s001.pdf]

Supplementary Figure S1

Alignment of Ctr9 homologues, made using MegAlign (DNAStar). Residues that are identical to the majority consensus are highlighted in black, and residues that are within the consensus group (consensus #1) in grey. Groups are f-hydrophobic; p-polar, a-acidic, b-basic. Organisms are: Tb- *T. brucei*, At- *Arabidopsis thaliana*, NP_178674.6; Dd- *Dictyostelium discoideum* XP_642310; Eh- *Entamoeba histolytica* EAL45089; Hs- *Homo sapiens* NP_055448; Ng- *Naegleria gruberi* XP_002679971; Sc- *Saccharomyces cerevisiae* Ctr9; Sp- *Schizosaccharomyces pombe* CAA15833.

```
      - - - - - - - - - - - - . . f p p f p f f f b . f p a a p - f a f f f . p f f .  Consensus #1
      - - - - - - - - - - - - - X X Q S I X I P X X X X X D X X - X E I D X D X L P D  Majority
                        10                  20                  30                  40
1     - - - - - - - - - - - - - - M Q Y I S E P V Q R L L D D S - T E A F T K C S V D  TbCtr9
1     - - - - - - - - - - - - - - M A S V Y I P V Q - N S E E E - V R V V L D Q L P R  AtCtr9
1     - - - - - - - - M D T K I Q S Q F I Y I P I R G F N D Q A - L K I D I S S L P -  DdCtr9
1     - - - - - - - - - M S E L V A L F A P E P I K L M I D P I R Y V I I P R P I T I  EhCtr9
1     - - - - - - - - - - - - M S R G S I E I P L R - D T D E V - I E L D F D Q L P -  HsCtr9
1     - - - - - - - - - - - - - S D N T S T A V I D I P T A D G - Y D V A V D L M N D  NgCtr9
1     T N A M K V E G Y P S M E W P T S L D I P L K A S E E L V - G I D L E T D L P D  ScCtr9
1     - - - - M E G F T L E T H A S R I I E V P L L G Q E D Q S - V E I D C S S L P S  SpTpr1

      a f . a f f . f f b . a . f f f a f f f . f f . a p p . p p b . a . f f . f f a  Consensus #1
      D A X X L K X I L X A E X A P L E L W L K X A L E Y Y K Q G K T E X F I K L L E  Majority
                        50                  60                  70                  80
26    D A V A M L D A A R R E - - - - - - - - - - - - - - - E K G T A E L H D V F V V  TbCtr9
25    D A S D I L D I L K A E Q A P L D L W L I I A R E Y F K Q G K I E Q F R Q I L E  AtCtr9
31    D S K E L I E V L K A E L A P L D L W L K L A N E Y Y K Q D R I T D F I E V L K  DdCtr9
32    Q F H K L K S Y F Q K D - - P I A T V Q V V A S L V R E Q R R E E A Q K F L L E  EhCtr9
26    E G D E V I S I L K Q E H T Q L H I W I A L A L E Y Y K Q G K T E E F V K L L E  HsCtr9
27    E Y D D I K N L L V D E K V P L E Y Y L K T A I L Y H N I D K T D H F N K L L N  NgCtr9
40    D P T D L K T I L V E E N S E K E H W L T I A L A Y C N H G K T N E G I K L I E  ScCtr9
36    D A T E L C E I L V N E Q A P R E F W T K F A H E Y Y I R G L R E Q A I L I L K  SpTpr1

      f f f a - - . a f a p p p . a . . . - - - - - - f f f f p f f f p . p f p . p -  Consensus #1
      S X X X - - X D L X Q Y Y Q D N X X - - - - - - I A A L N A L A A Y Y X X L G -  Majority
                        90                 100                 110                 120
51    S C M R - - - - - - - - - - - - - - - - - - - - - A S I A A M C G E R S L E G -  TbCtr9
65    E G S S - - S D I D E Y Y A D V K Y - - - - E R I A I L N A L G A Y Y S Y L G -  AtCtr9
71    Q V T D - - A D L I Q Y Y K D S K L - - - - D R I A M L N A L A S Y Y T Q L G -  DdCtr9
70    I M T N - - S S L S Q L S Q T I E R - - - - - - - Y F Y I I I A S L N A Y T G -  EhCtr9
66    A A R - - - I D G N L D Y R D H E K - - - - D Q M T C L D T L A A Y Y V Q Q A -  HsCtr9
67    L V I E D S E V E D K Y Y Q D N P K P L L D A R T D A L N I L V G H L I E Q Y N  NgCtr9
80    M A L D - - - - V F Q N S E R A S - - - - - - - L H T F L T W A H L N L A K G -  ScCtr9
76    S G L E T L K D S E S L C I L N A N - - - - - - I A A I Y L S M A R E A M L K -  SpTpr1

      - - - - - - - - - - - - - - . a . . . . . b a a p f p p f p . p f p b f . b f a  Consensus #1
      - - - - - - - - - - - - - - - X K X X X - X E E X L T X A T L Y L X K A X R I D  Majority
                       130                 140                 150                 160
69    - - - - - - - - - - - - - - - - - - - - - - - - - - - - - - - Y M A D A S A S P  TbCtr9
98    - - - - - - - - - - - - - K T E T K N R E K E E Q F I S A T R Y Y N K A S R I D  AtCtr9
104   - - - - - - - - - - - - - S Q E K D K S R R E E Y F S N A T F H F I K A D R I D  DdCtr9
100   - - - - - - - - - - - - - - - - - - N - - - E Q N N K I C H E Y L E K S K R F G  EhCtr9
98    - - - - - - - - - - - - - R K E K N K D N K K D L I T Q A T L L Y I M A D K I I  HsCtr9
107   K V R I H Q F D N E R Q A M E D I C D N K R E E L L N D I N K L L G K A E Q F N  NgCtr9
108   - - - - - - - - - - - - - - Q S L S V E T K E H E L T Q A E L N L K D A I G I D  ScCtr9
109   - - - - - - - - - - - - - - - - K D T D L R D E Q L R N V R T Y L E A A N N I D  SpTpr1
```

```
      f p . f . p f f p b . f f f f p b p - - - - - . f a p f . . p f . f f f . f . b  Consensus #1
      P X E P S N X L G X G V L L L X K G - - - - - S L D X A X S X F X F V L D X X K  Majority
                          170                 180                 190                 200
78    L S R P V L L Y L E G V A A M T S G - - - - T S L Q T A S M K F E E A L R V D K  TbCtr9
125   M H E P S T W V G K G Q L L L A K G - - - - - E I D N A L Q A F K I V L D T A P  AtCtr9
131   P H Q P L T W I G K A V L L L T K G - - - - - D Y E R A E S N F K Q V L D I A K  DdCtr9
119   D N E F A E C I Y G F N A F Y S K K - - - - - - - - P C K D Y F K R A L E L N K  EhCtr9
125   M Y D Q N H L L G R A C F C L L E G D - - - - K M D Q A D A Q F H F V L N Q S P  HsCtr9
147   P S K L S N F Y S R G V L H L N L G - - - - - A L D K A E S S F D Y I I T V D K  NgCtr9
134   P T W I G N M L A T V E L Y Y Q R G - - - - - H Y D K A L E T S D L F V K S I H  ScCtr9
133   S K S E I N V L L H G I Y R I L L N P T D K E S L E N A A R C F D F V L Q K S G  SpTpr1

      p p - - - - - - - - - - - - - f f f f f p . f p f . f p b b p p b . f f p . p . b f  Consensus #1
      X N - - - - - - - - - - - I X A L L G X A C I L F N K X N Y X E A L S X Y Q X A  Majority
                          210                 220                 230                 240
114   H F - - - - - - - - - - - I L A R M G L A A V C F Y M K D Y K R S F S H Y R A V  TbCtr9
160   D N - - - - - - - - - - - V P A L L G Q A S V E F N R G R F S E S L Q L Y K R A  AtCtr9
166   S N N T - - - - - - L P V L P A K L G S A C I L F N Q G N Y I K A L D T Y Q K V  DdCtr9
151   N Y - - - - - - - - - - - A P A L I G L A L C E L K D H K D G V A L S Y F I Q A  EhCtr9
161   N N - - - - - - - - - - - I P A L L G K A C I S F N K K D Y R G A L A Y Y K K A  HsCtr9
182   D N - - - - - - - - - - - I L S K L G M A C I K Y H K K Q Y K E A L S E F E Q C  NgCtr9
169   A E D H R S G R Q S K P N C L F L L L R A K L L Y Q K E N Y M A S L K I F Q E L  ScCtr9
173   G N - - - - - - - - - - - I L G F L G K A R I L Y A K G N Y R S A L K L Y Q R A  SpTpr1

      f . p p - f p p f f a f b f p f p f p f . . f p . f a b f . . f f a b f f p f a  Consensus #1
      L X T N - P X C P X D V R L G I G L C F Y K L G R L K K A X X A F E R X L Q L D  Majority
                          250                 260                 270                 280
143   L E T V G S S A P G I V R V G L G L C A Y R L G R M D A A V K W L E R A L A V N  TbCtr9
189   L Q V F - P G C P A A V R L G I G L C R Y K L G Q L D K A R Q A F D R V L Q L D  AtCtr9
200   I Q Q N - S N C L P S V R L G L G Y C Y F K L G R T K K A K E A F K R V L E L D  DdCtr9
180   L K K Q - P T N - V S I R C G I G R I Y Y S Q K E I K L A I R C Y E S A L V L D  EhCtr9
190   L R T N - P G C P A E V R L G M G H C F V K L N K L E K A R L A F S R A L E L N  HsCtr9
211   L L M N - P Q G P A D I R L G M G L C H Y Q L D N F E R A K Q C F E R V L Q L D  NgCtr9
209   L V I N - P V L Q P D P R I G I G L C F W Q L K D S K M A I K S W Q R A L Q L N  ScCtr9
202   L V S N - P Q F K P D P R I G I G L C F W N L D M K T D A L S A W T R V Q Q L D  SpTpr1

      f . p - - f a f f f p f . f f a f p f . . . - - - - - - - - . p f b b f f . - f f  Consensus #1
      P K N - - V X A L I G L A X X D L N F R X X - - - - - - - - X X I K K A L X - L L  Majority
                          290                 300                 310                 320
183   G E D - - V A A L L S L L V V Y L A Q R - - - - - - - - - - R I K E V I D - V V  TbCtr9
228   P D N - - V E A L V A L G I M D L Q A N D S - - - - - - - I G M R K G M D - R M  AtCtr9
239   R D N - - V E A M I G L A L V L M N E N - - - - - - - - - - Q I P E A M K - L I  DdCtr9
218   N I C - - M D A L I N L S R I Y W D I R T P - - - - - - - Q I V K R A L E - L A  EhCtr9
229   S K C - - V G A L V G L A V L E L N N K E A - - - - - - - D S I K N G V Q - L L  HsCtr9
250   P N N - - V S A L I Y L A I I D L N S R D E - - - - - - - E L I Q N A V K N Y L  NgCtr9
248   P K N T S A S I L V L L G E F R E S F T N S T N D K T F K E A F T K A L S - D L  ScCtr9
241   P K N T V V D T Y I G L Y Y Y D L A F Q N V N - N D S F V Q N Y G K A L Q - H I  SpTpr1
```

```
    p b f . p f . f p p f f f f p f f p p b f f f - - - - b p a p . . f . . f f f .  Consensus #1
    X R A X X I D P X N X X X L N X L A N H F F F - - - - K G X Y K K V E E L A X H  Majority
                        330                 340                 350                 360
210 R R L Q K V F P D N A M I L L K T T D I L Y F R A V S Q G R V K A L A P S I L H  TbCtr9
258 Q Q A F E I Y P Y C A S A L N Y L A N H F F F - - - - T G Q H F L V E Q L T E T  AtCtr9
266 L S A Y Q L A P I N S T V L N H L A N H Y F F - - - - R G E Y N K V N T L G V A  DdCtr9
248 N R A L M I D N K C A P A A I V L C E A A M K - - - - C N N Y K V V S E F A N V  EhCtr9
259 S R A Y T I D P S N P M V L N H L A N H F F F - - - - K K D Y S K V Q H L A L H  HsCtr9
281 K R A Y S L D P G N S Q V L N E L C N H F F F - - - - R R E V D K T E E L V E A  NgCtr9
287 N N I F S E N Q H N P V L L T L L Q T Y Y Y F - - - - K G D Y Q T V L D I Y H H  ScCtr9
279 Q R A F K T R N N D P V A S S I L E R Y V Y S - - - - K K N Y E G C I K L A E N  SpTpr1

    f f p p p . - - - . . . f b f a p p p p f p b f f b f . p a . a b f f f p p p .  Consensus #1
    A X X N T X - - - X E X I K A E S C Y X L G R A Y H A K G D X X K A F Q Y Y X Q  Majority
                        370                 380                 390                 400
250 L L S E A R P T A R V E D A A I A D Y Q E G R L L L A L G D F T R A R V L L E S  TbCtr9
294 A L A V T T - - - H G P T K S H S F Y N L A R S Y H S K G D F E K A G M Y Y M A  AtCtr9
302 A F N N T D - - - V A H I K A E S A Y L I G R A F H A T Q R W Q D A I Q Y Y H Q  DdCtr9
284 A I N Y G N - - - - E Q E K I Y G H F T L G R I A H Q K Q D F V K A K E E Y N I  EhCtr9
295 A F H N T E - - - V E A M Q A E S C Y Q L A R S F H V Q E D Y D Q A F Q Y Y Y Q  HsCtr9
317 A F H N T K - - - S P K I K A E S C Y N M A R A Y H H K K D Y D S A F K Y Y Y R  NgCtr9
323 R I L K M S P M I A K I V L S E S S F W C G R A H Y A L G D Y R K S F I M F Q E  ScCtr9
315 V I Q N S F - - - S S S L I A D G Y Y W M G R A Y H Q M G N N E K A M A S Y Q K  SpTpr1

    f f p . . . . - - - - f f f f b f p f p p f p f b . p a f a p f f f p f a . f f  Consensus #1
    A V Q K X P X - - - - F L L A R F G L G Q X Q I X X X D L X X A X X X F E X X L  Majority
                        410                 420                 430                 440
290 S M Q V L P N - - - - C L A A R V H Y A R L L F L L C R D V E A E Q L I L R I N  TbCtr9
331 A I K E T N N N P H E F V F P Y F G L G Q V Q L K L C F L K G S V F N F E K V L  AtCtr9
339 A V Q K N P D - - - - L Y L A Q F G L G Q I H I H N E D Y D K A I L C F E Q V L  DdCtr9
320 G Y L S D T K - - C M F P A L H Y R M A Q V L F R E K N Y I Q V E K I I M N C L  EhCtr9
332 A T Q F A S S S - - - F V L P F F G L G Q M Y I Y R G D K F N A S Q C F E K V L  HsCtr9
354 S V S R L W P - - - E Y T L A R Y G L G Q L Y I Q R N E I D K A V E E F E Q I L  NgCtr9
363 S L K K N E D - - - - N L L A K L G L G Q T Q I K N N L L E E S I I T F E N L Y  ScCtr9
352 A K A A D D R - - - - H L L S S V G I G Q I Q I L Q N D L T S A K L T F E R I A  SpTpr1

    b . p f p p p a . f . f f p p f p f b . p . p a b p - - - - - - - - - - - - - -  Consensus #1
    K V N P N N X E T L K I L G S L Y A H L X X X E K X - - - - - - - - - - - - - -  Majority
                        450                 460                 470                 480
326 A E H P N Q K E V L Q M L A A H A S H L G L H E R - - - - - - - - - - - - - - -  TbCtr9
371 E V Y P D N C E T L K A L G H L Y T Q L G Q N E K - - - - - - - - - - - - - - -  AtCtr9
375 S K Q P N N Y E T L Q I L G S L Y K H G S L Y K S N V K S T T T T T S T T T T T  DdCtr9
358 K Y N G G D F D V L K I L G F T E L K L N K I N E T - - - - - - - - - - - - - -  EhCtr9
369 K A Y P N N Y E T M K I L G S L Y A A S E D Q E K R D I - - - - - - - - - - - -  HsCtr9
391 K V D P E N L E T N L A L G N L Y A R K R D S K K - - - - - - - - - - - - - - -  NgCtr9
399 K T N E S L Q E L N Y I L G M L Y A G K A F D A K T A K N - - - - - - - - - - -  ScCtr9
388 E Q N Q S C F E A L V V L G C L H A S D S K P D L T K A R - - - - - - - - - - -  SpTpr1
```

```
     -------------------ff.pfbbfp.f.f...pfff. Consensus #1
     -------------------ALEYLKKATXLDPDDSNALIX Majority
              490       500       510       520
351  -------------------ALECSKLLTESVAPGDVISWT TbCtr9
396  -------------------ALEYMRKATKLDPRDAQAFVG AtCtr9
415  NNNININNNNNLSNEIINKIKNVLKKATELNPNDSSNWFE DdCtr9
384  --------------------IEYLEKASVFKIDYTIELIV EhCtr9
397  -------------------AKGHLKKVTEQYPDDVEAWIE HsCtr9
416  -------------------SLAYLKKVLKKDPENINALIR NgCtr9
428  -------TSAKEQSNLNEKALKYLERYLKLTIATKNQLVI ScCtr9
417  --------------MLLDRAFNLVGSSKLPRVVDSDLYIT SpTpr1

     fp.ffa.p.fp.ffaf.pbff.ff..------...f.f.f Consensus #1
     LXXLLEXXXXXXALXAYKXAL-ILEX------X-XIXVPX Majority
              530       540       550       560
372  LAAWCSRLNSEESAKLLTHVLHIYEELG-------TPVPM TbCtr9
417  LGELLISSDTGAALDAFKMAR-TLMK------KGGQEVPI AtCtr9
455  LGQVLESTEVSTALEAYEKGL-NLLK------KDGIVPSL DdCtr9
404  SSLLEE-KQPKKALEHYQRIN-------------EINSTV EhCtr9
418  LAQILEQTDIQGALSAYGTATRILQE------KVQADVPP HsCtr9
437  IGEHER-HQIQLALDSLKEALTIIEEG-----ETELVVTH NgCtr9
461  SRAYLVISQLYELQNQYKTSLDYLSKALEEMEFIKKEIPL ScCtr9
443  QARLWEKEDTKKSLGFLTRALDFLES-------AHMSVGP SpTpr1

     affppffff.p.fp.ffaf.apff.ffpff.--------b. Consensus #1
     EXLNNIAVLHXXLGXLIEAXEXFXKALSLA--------XX Majority
              570       580       590       600
405  KLRANKASMCGDIGTLQNILESELGEDFLS--------KP TbCtr9
450  EVLNDIGALHFEREEFESALENFKEALGDGIWISFLDEKE AtCtr9
488  EIQNNIAVLRHQKGLLVEAEQTYLDIIKQ----SGYQLNQ DdCtr9
430  EVLNNMGCCYYFIQELIKSKECFEKALNMD--------K- EhCtr9
452  EILNNVGALHFRLGNLGEAKKYFLASLDRAKAEAEHDEHY HsCtr9
471  ELYNNIAVHYYKLGKNTESEEYFKKALSLAECNVMDNLDD NgCtr9
501  EVLNNLACYHFINGDFIKADDLFKQAKAKVS-------DK ScCtr9
476  ELLNNIAVLQYHLGLIPEAHGNIIKAKSVIP-------DA SpTpr1

     p..fp-----------------------------fpffpp Consensus #1
     NLEXN-----------------------------VTLXYN Majority
              610       620       630       640
437  ELPVN----------------------------YVPIVYN TbCtr9
490  NLEQTGVSVLGYKDTGIFHRLIESGHSVDVPWNKVTTLFN AtCtr9
524  FKSIN-----------------------------ITSTYN DdCtr9
461  GSELR-----------------------------TTLLFN EhCtr9
492  YNAIS-----------------------------VTTSYN HsCtr9
511  LHQAIEVK-------------------------HLSLVYN NgCtr9
534  DESVN-----------------------------ITLEYN ScCtr9
509  NPEIS-----------------------------ILLDYN SpTpr1
```

```
     f f b f . a . f . . f a . f p . f p . p f f b . b f p p f a f p f b - - f p . f Consensus #1
     L A R L L E X M X D T X X A E X L Y X X I L K E H P X Y I D A Y L R - - L X X X Majority
                   650                 660                 670                 680
449  M A R L L E T S - D K V R A C E L Y S Y L V K H H P C F S Y P Y F R - - L Y E I TbCtr9
530  L A R L L E Q I H K T E A A T F M Y R L I L F K Y P G Y I D A Y L R - - L A A S AtCtr9
535  L A R L Y E T M G Q V N K A E E L Y K G I I K E H P N Y Y D C Y L R - - L S C I DdCtr9
472  K G R V L E E M K L W D E A N K C Y Q E I I K E N P W Y F D A R I R - - R G Y H EhCtr9
503  L A R L Y E A M C E G H E A E K L Y K N I L R E H P N Y V D C Y L R - - L G A M HsCtr9
526  F A R F K E V S K S L D D A Q K L Y L K I V A Q H P S Y I N A Y L R - - L G K I NgCtr9
545  I A R T N E K N - D C F K S E S I Y S Q V T S L H P A Y I A A R I R N L Y L K F ScCtr9
520  L A R C E E E I G N T S V A S E A Y V S I L E K H P S F I D A R I R - - K G L I SpTpr1

     f b . p p p . p a f . . f f b a f f p f . f p p f a f f f f f p . f f f . p . a Consensus #1
     A K X X G N Y X E A X E X X N E A L X X X P D N P E A W X L L G X L L L X Q K E Majority
                   690                 700                 710                 720
486  A K A E R C W R K A I M W M N L L R Q A I P D E P R A L V Y I C L L F F E Q R R TbCtr9
568  A K A Q N N L P L A I E L V N E A L K V D D K N P N A I S L L G E L E L K N D D AtCtr9
573  C K Q Q G D Y Y E A G E W I R E V L D I Q P D N Q E A W A L Y G N L H L Y K E E DdCtr9
510  L W D E K Q Y N L A S Q E L V E T I I N F P N C E D A K L L L G E M L C Q Q G K EhCtr9
541  A R D K G N F Y E A S D W F K E A L Q I N Q D H P D A W S L I G N L H L A K Q E HsCtr9
564  Q Q K N G N H E K A I H F C K L A T S L E P N N A A T W A F L G Q T Y L E Q N N NgCtr9
584  A Q S K I E D S D M S T E M N K L L D L N K S D L E I R S F Y G W Y L K N S K E ScCtr9
558  Q L S N - P N E E T F K E I R H I M N A D S Q N L E V R A F F G W Y L S K Q K R SpTpr1

     f . f f . b . f . . f p p p f - - - . . b p a . p f f f p f p - p f p f b p f b p Consensus #1
     W X P A Q K I F X H I T X X X - - - X K X D X Y A L L X L G - N V Y X X A A R Q Majority
                   730                 740                 750                 760
526  Y A A A M N I I R L A K T R - - - - - - S - C V V A L A L G - Q I Y L R H A Q Q TbCtr9
608  W V K A K E T F R - A A N D A - - T D G K D S Y A I L S L G N W N Y F A A M R N AtCtr9
613  W Y P A Q K N F E Q I T E N P - - D N K N E T Y A S L S L G - N I Y Y N A K F S DdCtr9
550  I D D A F K I F N S V T N H N - - - - R T N L Y A F L A L A - R V M A K Y G R Q EhCtr9
581  W G P G Q K K F E R I L K Q P - - S T Q S D T Y S M L A L G - N V W L Q T L H Q HsCtr9
604  Y T E A Q K A F E Y I T Q N - - - I D K N D I Y A L L G M G - N V Y F K S I R I NgCtr9
624  R K N N E K S T T H N K E T L V K Y N S H D A Y A L I S L A - N L Y V T I A R D ScCtr9
597  R P V E D P E V R H C S Q T L R - H W I D D I Y S L V Q L G - N A Y M R Q A R E SpTpr1

     . b - - - - - - b a . a b . . b . . . b f f . f p p b f f p f a f p p f p f f p p Consensus #1
     - K - - - - - - X E X X K X X K H L L K A X Q L Y X K V L X K D P X N X Y A A N G Majority
                   770                 780                 790                 800
558  H S - - - - - - - - - G D S Y R F L E L A K D R F H F A L Q K D K G N V L A A H G TbCtr9
645  E K - - - - - - R N P K L E A T H L E K A K E L Y T K V L I Q H N S N M Y A A N G AtCtr9
650  - - - - - - - - - - N P D K V E K Y I L N A E Q F Y N R V L T K N P T N I Y A A N G DdCtr9
585  - - - - - - - - - - - - D - - P K Q Q L E A V K L Y G K I L N A D P I N V L A L G G EhCtr9
618  P T - - - - - - R D R E K E K R H Q D R A L A I Y K Q V L R N D A K N L Y A A N G HsCtr9
640  A K P N P D E K E Q E R I E K H L D Y A I L F F E K T L K I D N S N M Y A A L N NgCtr9
663  G K K S R N P K E Q G K S K H S Y L K A I Q L Y Q K V L Q V D P F N I F A A Q G ScCtr9
635  F R V H N D - R E K L K R Q K L Y I K A I Q S Y D Q A I K F D P K N A H A A Q G SpTpr1
```

```
      f . f f f f a b p p f p . f f . f f p b f b a f f f - - - - - - . p f a f f f p  Consensus #1
      I X I X L A E K G X F X X A L X X F S Q V R E A L X - - - - - - D C X D V W X N  Majority
                         810                 820                 830                 840
590   M A C C L G L E G R H E S C L L L I D R V G E I V P - - - - - - N C S Y V R K H  TbCtr9
680   S G I V L A E K G Q F D I A K D V F I Q V Q E A A S G S - V F L Q M P D V W V N  AtCtr9
682   I G M I I A E K G N L N L A G E T F L Q I R E A S M - - - - - - D C I P V S V N  DdCtr9
613   L A L T T A E R K E N S S N P I P F N V I I E S L Q R V N D V C P S Y E V M F S  EhCtr9
653   I G A V L A H K G Y F R E A R D V F A Q V R E A T A - - - - - - D I S D V W L N  HsCtr9
680   S G C V L C E N G Y T E E G K A L I S R V R E I C V G D - D M K D I P E T Y I N  NgCtr9
703   L A I I F A E S K R L G P A L E I L R K V R D S L D - - - - - - - N E D V Q L N  ScCtr9
674   I A I I L A Q N R Q F S K A L L I I S K V R E A I K - - - - - - - D A T T L I N  SpTpr1
```

```
      f f b f p f . f b p f p . f f b f p a p p f b b f p p . - a p f . f f . p f f b  Consensus #1
      L A H C Y X E Q K Q F S X A I K X Y E X C L K K F X N - - E N P E V L X Y L A R  Majority
                         850                 860                 870                 880
624   Y E A H M A N A K I L S D S F K Q A I D Y L Q R D P Q - - R A P L Q S S S L A F  TbCtr9
719   L A H V Y F A Q G N F A L T V K M Y Q N C L R K F F Y N - T D S Q I L L Y L A R  AtCtr9
716   L A H I Y V S K G L F D N A I K L Y E G C L K K S T S P K E I E T I I M Y L A K  DdCtr9
653   M G T C Y Y N A H N I Q M A K S I F E S T L N Q Y G - - - E Q V E V L N G L A Q  EhCtr9
687   L A H I Y V E Q K Q Y I S A V Q M Y E N C L R K F Y K H - Q N T E V V L Y L A R  HsCtr9
719   L G H L A M I Q K Q F S Q A E K L Y S T C S K R F F N D - E N P M V L A Y L A K  NgCtr9
736   L A H C Y L E M R E Y G K A I E N Y E L V L K K F D N E K T R P H I L N L L G R  ScCtr9
707   I G N C L A E L K Q F S R A I E V F E T V Y S S T G E S - D T Y G V L S C L G R  SpTpr1
```

```
      . f f . . p b - - - - - - - f p a p f . p f b b f f . f . f p p f p - - - - - -  Consensus #1
      X L F D X X R - - - - - - - Y Q E X X X T L K K A L H L X P S N X S - - - - - -  Majority
                         890                 900                 910                 920
662   C L F C E G R - - - - - - - Y A D A I A V Q K K A V D E L P S E P - - - - - - -  TbCtr9
758   T H Y E A E Q - - - - - - - W Q E C K K T L L R A I H L T P S N Y I - - - - - -  AtCtr9
756   V Y F D A N R - - - - - - - F Y D S K Q T L K K A I H M Y P H N L S - - - - - -  DdCtr9
690   C E F M L N R - - - - - - - Y K E A I E H L E R A L K I E P V S - - - - - - - -  EhCtr9
726   A L F K C G K - - - - - - - L Q E C K Q T L L K A R H V A P S D T V - - - - - -  HsCtr9
758   S L F D N N K - - - - - - - H E E S I N I L K K I Q E Q D A T N L I - - - - - -  NgCtr9
776   A W Y A R A I K E T S V N F Y Q K A L E N A K T A L D L F V K E S S K S K F I H  ScCtr9
746   V W I Q R G R E S K N V D Y L K E S V R Y A T L A L E K N P E N P S - - - - - -  SpTpr1
```

```
      - f b f p f f f f f p . f f p . . f p . - - . . b p f p a f . p f f . p f a f f  Consensus #1
      - L X F N X A L V Y Q Q X A X X T L X K - - K K R T V X E V X N X X X E L E X A  Majority
                         930                 940                 950                 960
688   L L R F N L A L L Y C A S F V A S I S Q - K Q E Q S V Q E A K E L R S F L T E G  TbCtr9
785   - F R F D L G A V M Q K S S S S T L Q K - - K K R T A D E V R S T V A E A E N A  AtCtr9
783   - I H F N L A I S I E M Q A T I F L E K - - H Q K N A T E T F N I I K E L E F A  DdCtr9
715   - - - - Q I V Y N Y Q L V A S C Y L E E - - - - Q K Q K K K L Y K E E N E Q L R  EhCtr9
753   - L M F N V A L V L Q R L A T S V L K D - - E K S N L K E V L N A V K E L E L A  HsCtr9
785   - I K Y N I A L A F Y E K L V A T L N D - - S N K D L T K V Q I L E D Q N Q T I  NgCtr9
816   S V K F N I A L L H F Q I A E T L R R S N P K F R T V Q Q I K D S I E G L K E G  ScCtr9
780   - L L F N V A F V Q F Q I C E L I R Q K P E N S R T V E D L N F A M Q Q L D A S  SpTpr1
```

```
     f . f f p . f . . f p p f . b . - - - - - - f p p . . p . f . . f f . p f b p . Consensus #1
     X X L F T E L X K V X X L X X - - - - - - - A S T X E Q X X K K L L X T A K S H Majority
                        970                 980                 990                1000
727  L N I A H E F I K I E S E S R R - - - - - - L S E A K K L L K Q L C A Y C V H H TbCtr9
822  V R V F T Q L S A A S D L H V H G F D S K K I Q T H V Q Y C S H L L E A A K V H AtCtr9
820  Q R L I T P L A N T K S T P K L N F S P S K A K T H Q T S I E K I L V S L R I E DdCtr9
747  Q N V I G Y L Q Q F N N L K Y - - - - - - - - - - - - K - M K T F E E N W K S L EhCtr9
790  H R Y F S Y L S K V G D K M R - - F D L A L A A T E A R Q C S D L L S Q A Q Y H HsCtr9
822  I N L F S E I A E N T N S K K - - - - - - - N S C E F S L D E K I V V H K A K D NgCtr9
856  I E L F R E L N D L K E F N M I P - - - - - K E E L E Q R I Q L G E T T M K S A ScCtr9
819  I E T F T K L V S V E H P P Y S - - - - - - P T S I E Q R A K M A K N T T K R Q SpTpr1

     f a . f f . . . f a . b - - - - - - - - - - - - - - - . . . . b . f . a . a . - - - . b Consensus #1
     L E R A X K E Q E E X X - - - - - - - - - - - - - - - E X X A X X X E A E E - - - R Majority
                        1010                1020                1030               1040
761  N D L S I P K V V A N G - - - - - - - - - - - - - - - - - - - - - - - - - - - - TbCtr9
862  R E A A E Q E E L Q N R - - - - - - - - Q R L E V A R Q A A L A E E A R - - - R AtCtr9
860  H E S L V K I E A D L S K K R E A A F E E V K R L E E E K R I R D L E L K Q Q L DdCtr9
774  I E Q A R K D Q E E K Q - - - - - - - - - - - - - - - - R L I K E H E E - - Q R EhCtr9
828  V A R A R K Q D E E E R E L R - A K Q E Q E K E L L R Q K L L K E Q E E K R L R HsCtr9
855  F I T V L E T K I K E K - - - - - - - - - - - - I E K Y R E K A E K E E - - L A NgCtr9
891  L E R S L N E Q E E F K K - - - - - - - - - - - - E Q R A K I D E A R K - - I L ScCtr9
853  L E R A I Q A Q I E Y E K - - - - - - - - - - - - S V A A K I E D A R I Q - - R SpTpr1

     a b a . . b b p . f . b . . . a a . p . b f f . a p a a f . b f . a a . b . f . Consensus #1
     E X E E X K X X X L E K E E E E E E X X R L L X E Q E E X X R X K E E K X E G T Majority
                        1050                1060                1070               1080
773  - - - - - H R A A L E A E R Q D A E W C R V Y N E H L E Q K R M L E E R R L A D TbCtr9
891  K A E E Q R K Y Q L E K R K Q E E E L R R L K Q E E E K I Q R I K E Q W K S S T AtCtr9
900  E E K L E A E R K L A F E E E E K T R E E L A K N N A A A A M I D D D Y K D G E DdCtr9
796  K R K I E E E N Q R K K E E E E K E Q Q R R M E E K K M I I E K T K K E Q E M I EhCtr9
867  E K E E Q K K L L E Q R A Q Y V E K I K N I L M F T G E T E A T K E K K R G G G HsCtr9
881  R H E L K K K Q E D A F K I I E E Q I K K Q L E D Q E E L R R V K E E K L R E T NgCtr9
917  E E N E L K E Q G W M K Q E E E A R R L K L E K Q A E E Y R K I Q D E A Q K I F ScCtr9
879  E K E K A R R L A E E E A L L K E K Q E R E R Q L Q E E R Q K M Q E E V L E W R SpTpr1

     p p . . b . b - - - - - - - - . f . a a a a a a a p f . . b b - - - - - - - - - - - Consensus #1
     G G R R K X X - - - - - - - - X E D D X X E D X P E K K K - - - - - - - - - - - Majority
                        1090                1100                1110               1120
808  E R Q R R E Q - - - - - - - - - E Q Q V A G E I L E D F K - - - - - - - - - - - TbCtr9
931  P G S N K R K - - - - - - D R V E D D D G E S K P S E R R - - R K K - - - - - - AtCtr9
940  G G S T K K R K K N K K D Q Q Q Q D D D F D D Q P A E S K - - - - - - - - - - - DdCtr9
836  G K R I E E R - - - - - - - - - L K D V E E N I T E K K K - - - - - - - - - - - EhCtr9
907  G G R R S K K G - G E F D E F V N D D T D D D L P I S K K K K R R K G S G S E Q HsCtr9
921  F G R L E S I K A N L K T T A F E E D E D E E K K T K K R - - - - - - - - - - - NgCtr9
957  Q V R E A M A - - - - - - - I S E H N V K D D S D L S D K - - - - - - - - - - - ScCtr9
919  K S Q Q K A S E - - - - D D M S L S D D E E K Q S G K K K - - - - - - - - - - - SpTpr1
```

```
     - - - - - - - - - - b b . a b b a b . . . b a b p a a - - - - - - - - - - - - -  Consensus #1
     - - - - - - - - - - X K K X K K X K K X P K E K X E X - - - - - - - - - - - - -  Majority
                         1130                1140                1150                1160
828  - - - - - - - - - - - - - E L H G H R V P Q V K N E N - - - - - - - - - - - - -  TbCtr9
957  - - - - - - G G K R R K K D K S S R A R H Y E D D E E - E - - - - - - - - - - -  AtCtr9
969  - - - - - - - - K K Q K K E K K E K K E K K E K K E K - - - - - - - - - - - - -  DdCtr9
856  - - - - - - - - - - - K E D R K E K K E R K R K V L E - - - - - - - - - - - - -  EhCtr9
946  E G E D E E G G E R K K K K R R R H P K G E E G S D D D E T E N G P K P K K R R  HsCtr9
950  - - - - - - - - - - K K T Q D Y D D D L Q A E N N E N - - - - - - - - - - - - -  NgCtr9
979  - - - - - - - - - - - - D N E Y D E E Q P R Q K R K R - - - - - - - - - - - - -  ScCtr9
944  - - - - - - - - - - K K D R K K R K S K S K Q E S S D - - - - - - - - - - - - -  SpTpr1

     - - - - - - - - - - - - - - - - - - - - - - - - - - - - - - - - - - - - - - - -  Consensus #1
     - - - - - - - - - - - - - - - - - - - - - - - - - - - - - - - - - - - - - - - -  Majority
                         1170                1180                1190                1200
842  - - - - - - - - - - - - - - - - - - - - - - - - - - - - - - - - - - - - - - - -  TbCtr9
979  - - - - - - - - - - - - - - - - - - - - - - - - - - - - - - - - - A A T M D D H  AtCtr9
988  - - - - - - - - - - - - - - - - - - - - - - - - - - - - - - - - - - - - K K K S  DdCtr9
872  - - - - - - - - - - - - - - - - - - - - - - - - - - - - - - - - - - - - - - - -  EhCtr9
986  P P K A E K K K A P K P E R L P P S M K G K I K S K A I I S S S D D S S D E D K  HsCtr9
967  - - - - - - - - - - - - - - - - - - - - - - - - - - - - - - - - - - - - - - - -  NgCtr9
994  - - - - - - - - - - - - - - - - - - - - - - - - - - - - - - - - - - - - - - - -  ScCtr9
961  - - - - - - - - - - - - - - - - - - - - - - - - - - - - - - - - - - - - - - - -  SpTpr1

     - - - - - - - - - - - - - - - - - - - - - . - - - - - f . . . . a . a a . . . .  Consensus #1
     - - - - - - - - - - - - - - - - - - - - - X - - - - - X X X - X D X D E X K X X  Majority
                         1210                1220                1230                1240
842  - - - - - - - - - - - - - - - - - - - - - - - - - - - - - - - - - - E G F A E S  TbCtr9
986  N E V E D E D A N T N Y N R - - - - - E D E M T T Q E A E E P V D D D A H D L L  AtCtr9
992  K K V S G E G E E G A D D N N - - - - D T E Q Q Q E E M A E D G D H K E K K K K  DdCtr9
872  - - - - - - - - - - - - - - - - - - - - - - - - - - - - - - - - - - E E E E A D  EhCtr9
1026 L K I A D E G H P R N S N S N S D S D E D E Q R K K C A S S E S D S D E N Q N K  HsCtr9
967  - - - - - - - - - - - - - - - - - - - - T E Q Q E A P A K K K R K P K E P K K N  NgCtr9
994  - - - - - - - - - - - - - - - - - - - - - - - - - - - S T K T K N S G E S K R R  ScCtr9
961  - - - - - - - - - - - - - - - - - - - - - - - - - - - - S G V S E D D E I P L S  SpTpr1

     . f . b f p . . - - - - - - - - - - a . . . . b . . b f . b . . f . . a p a a a  Consensus #1
     K A X K K X K K - - - - - - - - - - D S D X X R X X X V R X X X E X X E S D E D  Majority
                         1250                1260                1270                1280
848  P A P W F S T S - - - - - - - - - - - - - - - V P Q D L V Q G E L R S M E D D D  TbCtr9
1021 A A A G L E D P - - - - - - - - D V D D D E V P T S G V R R R R A L S S S D E E  AtCtr9
1028 K K S K K D R K - - - - - - - D K K D K K Q K R K E K K Q R E K E E R E G E E D  DdCtr9
878  G E N K V S Y K - - - - - - - - - - - - - - - R K E R V N E K K M E E E K K E D  EhCtr9
1066 S G S E A G S P R R P R R Q R S D Q D S D S D Q P S R K R R P S G S E Q S D N E  HsCtr9
987  K E K K D K K E - - - - - - - T K K N S R L K K N A K A T E E E E R D E F D V D  NgCtr9
1007 K A A K K T L S - - - - - - - - - - D S D E D D D D V V K K P S H N K G K K S Q  ScCtr9
973  D A R N K T K K - - - - - - - - - - - R R V N R R V I S E E Y T E D Q D S D A E  SpTpr1
```

```
     p f p a . . a p b p f p a p p a p a . . . p . . p a . . f . . . p a a . a . . .  Consensus #1
     X V X E E E X N R X I E E S D X N E S X D X E A X D X X E X X - X D E X E X - -  Majority

                      1290                1300                1310                1320

873  A V L F S G D N F A G G V S G A G E D K S D T A N D G N I                        TbCtr9
1053 G E L M E E S H P N S S P Q K E K E E S N G E A G D P N M E E E E E E E E A N    AtCtr9
1061 D E N E E T E N R E E Q D D D N T E K V D Q E M Q D A K E A E K Q K E V E D F F  DdCtr9
903  N I E E K K E N H S I E Q S D D V V S L                                          EhCtr9
1106 S V Q S G R S H S G V S E N D S R P A S P S A E S D H E S E R G S D N E G S G Q  HsCtr9
1020 D V P Q S S S A R V I N D E N E N D N E E D E Y N E E G P S R A T D E G E E E F  NgCtr9
1037 L S N E F I E D S D E E E A Q M S G S E Q N K N D D N D E N N D N D D N D G L F  ScCtr9
1002 G N Q E E E V S R T I E E K Q D N D I T D N Q D D N K E L N L F S E E D E E      SpTpr1

     . . . . . . . . . . . . . . . . . . . . . . . . . . . . .                        Consensus #1
     - - - - - - - - - - - - - - - - - - - - - - - - - - - - -                        Majority

                      1330                1340

901                                                                                   TbCtr9
1091                                                                                  AtCtr9
1101 G S E N E M                                                                      DdCtr9
922                                                                                   EhCtr9
1146 G S G N E S E P E G S N N E A S D R G S E H G S D D S D                          HsCtr9
1060 A F D E                                                                          NgCtr9
1076                                                                                  ScCtr9
1039                                                                                  SpTpr1
```

Consensus 'Consensus #1': When 50% (4) match the residue group of the Consensus show the group label of the Consensus, otherwise show '.'. Residue Groupings of Consensus #1 are: a=(DE), b=(HKR), f=(AFILMPVW), p=(CGNQSTY).

Decoration 'Decoration #1': Shade (with black at 50% fill) residues that match the consensus named 'Consensus #1' exactly.

Decoration 'Decoration #2': Shade (with solid black) residues that match the Consensus exactly.
