# Supplementary material for: Depletion of Trypanosome CTR9 Leads to Gene Expression Defects
Source: PLoS One. 2012 Apr 20;7(4):e34256. doi: 10.1371/journal.pone.0034256 (PMC3332058; doi:10.1371/journal.pone.0034256)
Supplement: Figure S2 — Alignment of Cdc73 homologues, made using MegAlign (DNAStar). Details as for Figure S1. Organisms are: Tb- T. brucei, Sc- Saccharomyces cerevisiae; Sp- Schizosaccharomyces pombe NP_595891.1; Hs- Homo sapiens NP_078805.3; Dd- Dictyostelium discoideum XP_644100.3; Ng- Naegleria gruberi XP_002683436.1; Pf- Plasmodium falciparum XP_001351765.1; Tt- Tetrahymena thermophila EAR84002.1; Pi- Phytophthora infestans XP_002903756.1; Eh- Entamoeba histolytica XP_648719.1; Tv- Trichomonas vaginalis XP_001310227.1; Tp- Thalassosira pseudonana XP_002287950.1. (PDF) [file pone.0034256.s002.pdf]

Supplementary Figure S2

Alignment of Cdc73 homologues, made using MegAlign (DNAStar). Details as for Figure S1. Organisms are: Tb- *T. brucei*, Sc- *Saccharomyces cerevisiae*; Sp- *Schizosaccharomyces pombe* NP_595891.1; Hs- *Homo sapiens* NP_078805.3; Dd- *Dictyostelium discoideum* XP_644100.3; Ng- *Naegleria gruberi* XP_002683436.1; Pf- *Plasmodium falciparum* XP_001351765.1; Tt- *Tetrahymena thermophila* EAR84002.1; Pi- *Phytophthora infestans* XP_002903756.1; Eh- *Entamoeba histolytica* XP_648719.1; Tv- *Trichomonas vaginalis* XP_001310227.1; Tp- *Thalassosira pseudonana* XP_002287950.1.

```
     - - - - - - - - - - - - - - - - - - - - - - - - - - - - - - - - - - - - - - - -  Consensus #1
     - - - - - - - - - - - - - - - - - - - - - - - - - - - - - - - - - - - - - - - -  Majority
                       10                  20                  30                  40
1    - - - - - - - - - - - - - - - - - - - - - - - - - - - - - - - - - - - - - - - -  TbCdc73
1    - - - - - - - - - - - - - - - - - - - - - - - - - - - - - - - - - - - - - - - -  ScCdc73
1    - - - - - - - - - - - - - - - - - - - - - - - - - - - - - - - - - - - - - - - -  SpCdc73
1    - - - - - - - - - - - - - - - - - - - - - - - - - - - - - - - - - - - - - - - -  HsCdc73
1    - - - - - - - - - - - - - - - - - - - - - - - - - - - - - - - - - - - - - - - -  DdCdc73
1    - - - - - - - - - - - - - - - - - - - - - - - - - - - - - - - - - - - - - - - -  NgCdc73
1    M E N K I M L N I K V L M K K F L T E E K E Y I K F M N K E K N D I I V F E K E  PfCdc73
1    - - - - - - - - - - - - - - - - - - - - - - - - - - - - - - - - - - - - - - - -  TtCdc73
1    - - - - - - - - - - - - - - - - - - - - - - - - - - - - - - - - - - - - - - - -  PiCdc73
1    - - - - - - - - - - - - - - - - - - - - - - - - - - - - - - - - - - - - - - - -  EhCdc73
1    - - - - - - - - - - - - - - - - - - - - - - - - - - - - - - - - - - - - - - - -  TvCdc73
1    - - - - - - - - - - - - - - - - - - - - - - - - - - - - - - - - - - - - - - - -  TpCdc73

     - - - - - - - - - - - - - - - - - - - - - - - - - - - - - - - - - - - - - - - -  Consensus #1
     - - - - - - - - - - - - - - - - - - - - - - - - - - - - - - - - - - - - - - - -  Majority
                       50                  60                  70                  80
1    - - - - - - - - - - - - - - - - - - - - - - - - - - - - - - - - - - - - - - - -  TbCdc73
1    - - - - - - - - - - - - - - - - - - - - - - - - - - - - - - - - - - - - - - - -  ScCdc73
1    - - - - - - - - - - - - - - - - - - - - - - - - - - - - - - - - - - - - - - - -  SpCdc73
1    - - - - - - - - - - - - - - - - - - - - - - - - - - - - - - - - - - - - - - - -  HsCdc73
1    - - - - - - - - - - - - - - - - - - - - - - - - - - - - - - - - - - - - - - - -  DdCdc73
1    - - - - - - - - - - - - - - - - - - - - - - - - - - - - - - - - - - - - - - - -  NgCdc73
41   N L Y I Y T N V L C G I E S R K K E K Y N V G D I Y L F L C L A K S N Y T F S Y  PfCdc73
1    - - - - - - - - - - - - - - - - - - - - - - - - - - - - - - - - - - - - - - - -  TtCdc73
1    - - - - - - - - - - - - - - - - - - - - - - - - - - - - - - - - - - - - - - - -  PiCdc73
1    - - - - - - - - - - - - - - - - - - - - - - - - - - - - - - - - - - - - - - - -  EhCdc73
1    - - - - - - - - - - - - - - - - - - - - - - - - - - - - - - - - - - - - - - - -  TvCdc73
1    - - - - - - - - - - - - - - - - - - - - - - - - - - - - - - - - - - - - - - - -  TpCdc73

     - - - - - - - - - - - - - - - - - - - - - - - - - - - - - - - - - - - - - - - -  Consensus #1
     - - - - - - - - - - - - - - - - - - - - - - - - - - - - - - - - - - - - - - - -  Majority
                       90                 100                 110                 120
1    - - - - - - - - - - - - - - - - - - - - - - - - - - - - - - - - - - - - - - - -  TbCdc73
1    - - - - - - - - - - - - - - - - - - - - - - - - - - - - - - - - - - - - - - - -  ScCdc73
1    - - - - - - - - - - - - - - - - - - - - - - - - - - - - - - - - - - - - - - - -  SpCdc73
1    - - - - - - - - - - - - - - - - - - - - - - - - - - - - - - - - - - - - - - - -  HsCdc73
1    - - - - - - - - - - - - - - - - - - - - - - - - - - - - - - - - - - - - - - - -  DdCdc73
1    - - - - - - - - - - - - - - - - - - - - - - - - - - - - - - - - - - - - - - - -  NgCdc73
81   I N S I G Y K Y I S I L E R N K I I K T I E E N Q E D T D E I K I N C Y I Y D Q  PfCdc73
1    - - - - - - - - - - - - - - - - - - - - - - - - - - - - - - - - - - - - - - - -  TtCdc73
1    - - - - - - - - - - - - - - - - - - - - - - - - - - - - - - - - - - - - - - - -  PiCdc73
1    - - - - - - - - - - - - - - - - - - - - - - - - - - - - - - - - - - - - - - - -  EhCdc73
1    - - - - - - - - - - - - - - - - - - - - - - - - - - - - - - - - - - - - - - - -  TvCdc73
1    - - - - - - - - - - - - - - - - - - - - - - - - - - - - - - - - - - - - - - - -  TpCdc73
```

```
     - - - - - - - - - - - - - - - - - - - - - - - - - - - - - - - - - - - - - - - -  Consensus #1
     - - - - - - - - - - - - - - - - - - - - - - - - - - - - - - - - - - - - - - - -  Majority
                       130                 140                 150                 160
1    - - - - - - - - - - - - - - - - - - - - - - - - - - - - - - - - - - - - - - - -  TbCdc73
1    - - - - - - - - - - - - - - - - - - - - - - - - - - - - - - - - - - - - - - - -  ScCdc73
1    - - - - - - - - - - - - - - - - - - - - - - - - - - - - - - - - - - - - - - - -  SpCdc73
1    - - - - - - - - - - - - - - - - - - - - - - - - - - - - - - - - - - - - - - - -  HsCdc73
1    - - - - - - - - - - - - - - - - - - - - - - - - - - - - - - - - - - - - - - - -  DdCdc73
1    - - - - - - - - - - - - - - - - - - - - - - - - - - - - - - - - - - - - - - - -  NgCdc73
121  E K N I N L N K Y I N T H N D Y L I N I D L F D K L D D V E M E S D I L F T Q K  PfCdc73
1    - - - - - - - - - - - - - - - - - - - - - - - - - - - - - - - - - - - - - - - -  TtCdc73
1    - - - - - - - - - - - - - - - - - - - - - - - - - - - - - - - - - - - - - - - -  PiCdc73
1    - - - - - - - - - - - - - - - - - - - - - - - - - - - - - - - - - - - - - - - -  EhCdc73
1    - - - - - - - - - - - - - - - - - - - - - - - - - - - - - - - - - - - - - - - -  TvCdc73
1    - - - - - - - - - - - - - - - - - - - - - - - - - - - - - - - - - - - - - - - -  TpCdc73

     - - - - - - - - - - - - - - - - - - - - - - - - - - - - - - - - - - - - - - - -  Consensus #1
     - - - - - - - - - - - - - - - - - - - - - - - - - - - - - - - - - - - - - - - -  Majority
                       170                 180                 190                 200
1    - - - - - - - - - - - - - - - - - - - - - - - - - - - - - - - - - - - - - - - -  TbCdc73
1    - - - - - - - - - - - - - - - - - - - - - - - - - - - - - - - - - - - - - - - -  ScCdc73
1    - - - - - - - - - - - - - - - - - - - - - - - - - - - - - - - - - - - - - - - -  SpCdc73
1    - - - - - - - - - - - - - - - - - - - - - - - - - - - - - - - - - - - - - - - -  HsCdc73
1    - - - - - - - - - - - - - - - - - - - - - - - - - - - - - - - - - - - - - - - -  DdCdc73
1    - - - - - - - - - - - - - - - - - - - - - - - - - - - - - - - - - - - - - - - -  NgCdc73
161  N Y N N D I Y D K F N Q D K Q N N D E Y I S N I N D K I I E K K N I N E D T H N  PfCdc73
1    - - - - - - - - - - - - - - - - - - - - - - - - - - - - - - - - - - - - - - - -  TtCdc73
1    - - - - - - - - - - - - - - - - - - - - - - - - - - - - - - - - - - - - - - - -  PiCdc73
1    - - - - - - - - - - - - - - - - - - - - - - - - - - - - - - - - - - - - - - - -  EhCdc73
1    - - - - - - - - - - - - - - - - - - - - - - - - - - - - - - - - - - - - - - - -  TvCdc73
1    - - - - - - - - - - - - - - - - - - - - - - - - - - - - - - - - - - - - - - - -  TpCdc73

     - - - - - - - - - - - - - - - - - - - - - - - - - - - - - - - - - - - - - - - -  Consensus #1
     - - - - - - - - - - - - - - - - - - - - - - - - - - - - - - - - - - - - - - - -  Majority
                       210                 220                 230                 240
1    - - - - - - - - - - - - - - - - - - - - - - - - - - - - - - - - - - - - - - - -  TbCdc73
1    - - - - - - - - - - - - - - - - - - - - - - - - - - - - - - - - - - - - - - - -  ScCdc73
1    - - - - - - - - - - - - - - - - - - - - - - - - - - - - - - - - - - - - - - - -  SpCdc73
1    - - - - - - - - - - - - - - - - - - - - - - - - - - - - - - - - - - - - - - - -  HsCdc73
1    - - - - - - - - - - - - - - - - - - - - - - - - - - - - - - - - - - - - - - - -  DdCdc73
1    - - - - - - - - - - - - - - - - - - - - - - - - - - - - - - - - - - - - - - - -  NgCdc73
201  Y T D E N L I S F D Y T K K L F D I N K I N E S F I I H M K N D D I F F C T S L  PfCdc73
1    - - - - - - - - - - - - - - - - - - - - - - - - - - - - - - - - - - - - - - - -  TtCdc73
1    - - - - - - - - - - - - - - - - - - - - - - - - - - - - - - - - - - - - - - - -  PiCdc73
1    - - - - - - - - - - - - - - - - - - - - - - - - - - - - - - - - - - - - - - - -  EhCdc73
1    - - - - - - - - - - - - - - - - - - - - - - - - - - - - - - - - - - - - - - - -  TvCdc73
1    - - - - - - - - - - - - - - - - - - - - - - - - - - - - - - - - - - - - - - - -  TpCdc73
```

```
    - - - - - - - - - - - - - - - - - - - - - - - - - - - - - - - - - - - - - - - - Consensus #1
    - - - - - - - - - - - - - - - - - - - - - - - - - - - - - - - - - - - - - - - - Majority
                      250                 260                 270                 280
1   - - - - - - - - - - - - - - - - - - - - - - - - - - - - - - - - - - - - - - - - TbCdc73
1   - - - - - - - - - - - - - - - - - - - - - - - - - - - - - - - - - - - - - - - - ScCdc73
1   - - - - - - - - - - - - - - - - - - - - - - - - - - - - - - - - - - - - - - - - SpCdc73
1   - - - - - - - - - - - - - - - - - - - - - - - - - - - - - - - - - - - - - - - - HsCdc73
1   - - - - - - - - - - - - - - - - - - - - - - - - - - - - - - - - - - - - - - - - DdCdc73
1   - - - - - - - - - - - - - - - - - - - - - - - - - - - - - - - - - - - - - - - - NgCdc73
241 Y N K Y K M R K S S K K K I N K L K K R K Y N F I Q D D V C S T I Y D E M V Y F PfCdc73
1   - - - - - - - - - - - - - - - - - - - - - - - - - - - - - - - - - - - - - - - - TtCdc73
1   - - - - - - - - - - - - - - - - - - - - - - - - - - - - - - - - - - - - - - - - PiCdc73
1   - - - - - - - - - - - - - - - - - - - - - - - - - - - - - - - - - - - - - - - - EhCdc73
1   - - - - - - - - - - - - - - - - - - - - - - - - - - - - - - - - - - - - - - - - TvCdc73
1   - - - - - - - - - - - - - - - - - - - - - - - - - - - - - - - - - - - - - - - - TpCdc73

    - - - - - - - - - - - - - - - - - - - - - - - - - - - f f . f f . . f b . . f . Consensus #1
    - - - - - - - - - - - - - - - - - - - - - - - - - - - M A D I L X L L R K X L X Majority
                      290                 300                 310                 320
1   - - - - - - - - - - - - - - - - - - - - - - - - - - - - - - - - - - - - - - - - TbCdc73
1   - - - - - - - - - - - - - - - - - - - - - - - - - - - M A N S L D R L R E H L K ScCdc73
1   - - - - - - - - - - - - - - - - - - - - - - - - - - - - M D S L L L L K K S I A SpCdc73
1   - - - - - - - - - - - - - - - - - - - - - - - - - - - M A D V L S V L R Q Y N I HsCdc73
1   - - - - - - - - - - - - - - - - - - - - - - - - - - - M A D P L T I L R G I L L DdCdc73
1   - - - - - - - - - - - - - - - - - - - - - - - - - - - M D D I L E Q L N N T I K NgCdc73
281 N N N E I N T I I E G N N N V N N N F D F I K V E H N M E R I M N K T N Y D L Y PfCdc73
1   - - - - - - - - - - - - - - - - - - - - - - - - - - - - - - M I R N K R P N K I TtCdc73
1   - - - - - - - - - - - - - - - - - - - - - - - - - - - M A S K E D T P L G A L R PiCdc73
1   - - - - - - - - - - - - - - - - - - - - - - - - - - - - - - - - - - - - - - - - EhCdc73
1   - - - - - - - - - - - - - - - - - - - - - - - - - M S S L G P L Q L L R K W I E TvCdc73
1   - - - - - - - - - - - - - - - - - - - - - M P S T A A A S P I I P L L Q K A C A TpCdc73

    . . . . f . . - - - - - - - - - - - - - - - - - - - - - - - - - - - - - - - - - Consensus #1
    E X K X I X - - - - - - - - - - - - - - - - - - - - - - - - - - - - - - - - - - Majority
                      330                 340                 350                 360
1   - - - - - - - - - - - - - - - - - - - - - - - - - - - - - - - - - - - - - - - - TbCdc73
14  N G D K L V - - - - - - - - - - - - - - - - - - - - - - - - - - - - - - - - - - ScCdc73
13  E K K D V K - - - - - - - - - - - - - - - - - - - - - - - - - - - - - - - - - - SpCdc73
14  Q K K E I V V K G D E V I F G E F S W P K N V K T N Y V V W G T G K E G Q P R E HsCdc73
14  E G G T P N I E G D D Y S L G R Y K F N K N T P T A F K S S - - - - - - - T G S DdCdc73
14  S N T P I I N D N T N I I F G E K Q Y P I N G Q V K F Q S S - - - - - - - T - - NgCdc73
321 N D N Q K N N M D D H T Y N N D L Y Y Y N D N N V S D E N E K K L K T Y N L V K PfCdc73
11  D P L E L L R - - - - - - - - - - - - - - - - - - - - - - - - - - - - - - - - - TtCdc73
14  A L R A H L I A G - - - - - - - - - - - - - - - - - - - - - - - - - - - - - - - PiCdc73
1   - - - - - - - - - - - - - - - - - - - - - - - - - - - - - - - - - - - - - - - - EhCdc73
16  E H K T I D - - - - - - - - - - - - - - - - - - - - - - - - - - - - - - - - - - TvCdc73
20  A S L P T T T S L S P D G Q T L T I L S T P H D A T A Q L T L T L P D N K T V T TpCdc73
```

- Consensus #1
- Majority
370 380 390 400
1 - TbCdc73
20 - ScCdc73
19 - SpCdc73
54 Y Y T L D S I L F L L N N V H L S H P V Y Y V R R A A T E N I P V V R R P D R K D HsCdc73
47 N Y S L G F S L F F T F K S Y F K Y G A Y I L A A S R S N I Q A V N F N D K K E DdCdc73
45 - L T L G Q V W I C L K C I N D D Y S V Y L K Q C K E A N L N P V N I G I K N L NgCdc73
361 H Y Y E N N V F K N I F F Q N M N L Y S L I K N I Y L T N I S N E E Y I D K S T PfCdc73
18 - TtCdc73
23 - PiCdc73
1 - EhCdc73
22 - TvCdc73
60 Y S L A S I Y L Q L L H P G V L Q Y R K A C E G A G - - V E D P V K V L D K A G TpCdc73

f . . . - . . . . . . f . . . . . f . . f . f . . Consensus #1
X - T S X X E K X X X X X D X K F D G Majority
410 420 430 440
1 - M S L R E D W G R L V D E V C N M TbCdc73
20 L K N N E - - - - - - - - - - - - - - - G Q S T D D I T K A T M V E T L S S D G ScCdc73
19 L L A S - - - - - - - - - - - - - - - - S E A T S K V E K I E D A Q Y I L F D E SpCdc73
94 L L G Y L N G - - - - - - - E A S T S A S I D R S A P L E I G L Q R S T Q V K R HsCdc73
87 L L S Y L D G G E - I T A S I Q F D S L P T S T P T M L G S I S T A D D T I T I DdCdc73
84 L K S Y F - - - - - - - - - - - - - - - N V Q D T S L F K T S T T G E V K Q T K NgCdc73
401 H F I I Q N F Y N N F I Y Y I E N Y D K Y V L M S N E H R Q E Q I K N I K N N N PfCdc73
18 - E F T I N K K D V V K K D Q Y L F TtCdc73
23 - K P V E V E G E N L V F R D A S G PiCdc73
1 - EhCdc73
22 - D S D P G F I S TvCdc73
98 I T S F F L D A P - - - - - - - S - - - - D G G V D G A E G E G T A T P A K L D G TpCdc73

. . p a . f . . . . p p . f p f . . f f f p . . p . . . - - - - Consensus #1
X D G E S L X X E X K T R X K X K E G S K L S L R L X X L S X X X X X - - - - - Majority
450 460 470 480
18 D S T E P L P E E F V P S E F L P G G C E L P A D L I H Y L R S G A N - - - - - TbCdc73
45 S T Q D S F P L N E E T E I E I D G - S L V Q L R I I V H C W M N K D S - - - - ScCdc73
43 N T S P I L I D E P T K F I K L E N D S H F S L R S V Y F A W L L R D T - - - - SpCdc73
127 A A D E V L A E A K K P R I E D E E C V R L D K E R L A A R L E G H K E G - I V HsCdc73
126 P E G Q S I Q Q M D Q D S Q K Q S D E T T S S T Q L P F G G S L T Q K E Q E Q I DdCdc73
109 R K R E S T N D D D K K R L K P F D Y T K I T I P E K E F G E K E Q E S K L E F NgCdc73
441 T N G Q E K D I I N D E Y Y F N D E Y V D K N K L E V L L S S F N E N Y V D I N PfCdc73
35 F D Q T K L E L K T P T A W K K N T G G Y Y I L G D L W L F N Q K V Q Q N - - - TtCdc73
40 S E L R R L P K H A P T A Y H S K K L D K S Y D L L A V H T C F K H A D - - - - PiCdc73
1 - EhCdc73
30 V D G E K I D L E S E A N Y T P K K G S K L S M R T V Y L S L T T D R - - - - - TvCdc73
128 D D M D M S D E E A K E R E Q Q K E A S S R E E K N R R R S G S D S R H N K D K TpCdc73

- - - - - . p f p a p f p p p p . f . f p p . p . f . . . . f f p . f p p . - - Consensus #1
- - - - - - S F S D Y X S Q C Q X X A I X N V X F X D R X X L I X Y L X G K - - Majority
490 500 510 520
53 - - - - - - A F E A E T A D L F Q P Q T S T E E G F H A A P I V S R Y G - - - - TbCdc73
80 - - - - - - S A A D Y L A D C Q N K Q L T N V S F L Q R T D L I N W L S G N - - ScCdc73
79 - - - - - - S I A F Y I Q Q C S E L G I Q N L T F L E R T D L I S W L E G S - - SpCdc73
166 Q T E Q I R S L S E A M S V E K I A A I K A K I M A K K R S T I K T D L D D D I HsCdc73
166 D Q Q Q Q Q Q Q S S I Q Q Q Q Q D L G L S T Q Q Q P Q Q P Q Q E D D N N D Q - - DdCdc73
149 I K F L S N S L T D D I S N V N T E P F L P Y L E F D R P Q V L N Y I N N K E I NgCdc73
481 - E I Y K D N I S P S L E N Y K D M Y I N N N N N S N N M D V I P L L I R R K R PfCdc73
72 - - - - - M S M K H Y F N Q R Q S F A L E G I D K I D E D E I S N Y L Q G K I D TtCdc73
76 - - - - - L S F S D Y V L K C R E D K A A M V S T V D K K E L V A F L K G D - - PiCdc73
1 - EhCdc73
65 - - - - - - - F V D Y V K Q C Q A A K I N F M N F P D Y K A I T S Y L H S K S D TvCdc73
168 D R R R S K S K T P S K S D K K L V A A K Q S T P I T N E Q L V A N L S I I V D TpCdc73

- - - - - - p . . . f . . a f . . p b p a p p b . . . f - - - - - - - - - - - - Consensus #1
- - - - - - X X S X X I L X X K X Q K G E A S K - - - - - - - - - - - - - - - - Majority
530 540 550 560
83 - - - - - - - T A G T L R N L V G D R G E N P R - - - - - - - - - - - - - - - - TbCdc73
112 - - - - - - I E S S Q Y L K A P G Q K G E T S D K V D L - - - - - - - - - - - - ScCdc73
111 - - - - - - S D S E H I I G L E K P K P E G S - - - - - - - - - - - - - - - - - SpCdc73
206 T A L K - - Q R S F V D A E V D V T R D I V S R E R V W - - - - - - - - - - - - HsCdc73
204 - - - - - - Y Q D M A I Q E F D E F K S D Q I K I D P L - - - - - - - - - - - - DdCdc73
189 P F S T R I T Q T Q I E R E F V D K K G E N T K Q L S Y - - - - - - - - - - - - NgCdc73
520 K Y H N L S F D D K Y S L Q Y L K N F N D H D K L I F F N N M I S K Y I Y A I S PfCdc73
107 - - - - - - N S D K I D Q E A K Q Q L L S S R K S K P A - - - - - - - - - - - - TtCdc73
109 - - - - - I D S S P Q I L D A S G K P T A A S G D K D R - - - - - - - - - - - - PiCdc73
1 - - - - - - - - M T S T T D L K E Q Y E Q A N - - - - - - - - - - - - - - - - - EhCdc73
98 - - - - - - V L E G L I T D N K E E E A Q P A E E Q Q V - - - - - - - - - - - - TvCdc73
208 K R N K S E G K E A A S G D G G A S A S A A G D D S V K - - - - - - - - - - - - TpCdc73

- . . Consensus #1
- X - Majority
570 580 590 600
100 - TbCdc73
134 - E N ScCdc73
128 - SpCdc73
232 - R T HsCdc73
226 - L I DdCdc73
217 - F T NgCdc73
560 D K H F Y E R N K K T I N F V H N F L Y Q Y N V S L N D I K E Y Y N I I L T Q K PfCdc73
129 - E S TtCdc73
132 - K P PiCdc73
16 - EhCdc73
120 - P V TvCdc73
236 - E S TpCdc73

```
    . . . . . p . . . . . . p p f . . . . . f . . . f b a a - - - - - - - - - - - - Consensus #1
    - - L X - - X T X K E X T X X X X A K E V D E X I K E E - - - - - - - - - - - - Majority
                      610                 620                 630                 640
100 - - - - - - L P R H E G P N F Y E A Y R A H A A L N G E - - - - - - - - - - - - TbCdc73
136 K T L A - G E L S T V K S T T S A S L E N D S E V S D P - - - - - - - - - - - - ScCdc73
128 - - - - - - - - - - - - T D A A T S M D V D L H K K S E - - - - - - - - - - - - SpCdc73
234 R T T I L Q S T G K N F S K N I F A I L Q S V K A R E E - - - - - - - - - - - - HsCdc73
228 H D R E L I A Q Q Q K D K S I F A E K I D T K K I K T E S P L T V D T Y I E - - DdCdc73
219 S Y L E N I K R C K T H F T Q H K K D E K Q K R I E R E - - - - - - - - - - - - NgCdc73
600 K C L A P Q K I S Y E N E G H K S D N E F D D N D D D D N M F E I C K D Q N V D PfCdc73
131 S A L E G S L T K K I K T N D G E A K T V E M Q Q R E E Y L N - - - - - - - - - TtCdc73
134 A A K K T R E P E D E A R A A K K R K L A Q E K E N H R - - - - - - - - - - - - PiCdc73
16  - - - - - - - K M K I A T L P E Q I T P I S E E I K E K - - - - - - - - - - - - EhCdc73
122 A P V V E T Q V P Q P V Q Q E V R P E P V V V E S S Q T N - - - - - - - - - - - TvCdc73
238 D G T P A D I T P A T T P L P F D G E H D A E S E K E R E L L L S W L S P A G F TpCdc73

    - - - - - . f . . . f b . . f p . . p b . p f f b p - - - - - - - - - - - - - - Consensus #1
    - - - - - X V A X E F K N E R T X X N R N S A L K G - - - - - - - - - - - - - - Majority
                      650                 660                 670                 680
122 - - - - - - - - G G G R N N L T S S A S A A A A A G - - - - - - - - - - - - - - TbCdc73
163 - - - - - V V V E T M K H E R I L V D H N S A L R G - - - - - - - - - - - - - - ScCdc73
144 - - - - - E V N W L F E N T R T V S N H N S V L H G - - - - - - - - - - - - - - SpCdc73
262 - - - - - G R A P E Q R P A P N A A P V D P T L R T K Q P I P A A Y N - R - - - HsCdc73
266 - - - - - Q V K T K R K N M A T D D N R S S F I K A D I E V T K A I N N R - - - DdCdc73
247 - - - - - Q I A K Q Q G Y S Q E N A K L N L K I K G T F N E R N L W N S E - - - NgCdc73
640 L N N D N Y V D D V I N R N I K R Y N K K K I A K N K Y L Q I N S C D L N N S S PfCdc73
162 - - - - - A I S W I M K T E K P L T S R T R M L R S - - - - - - - - - - - - - - TtCdc73
162 - - - - - H K H G E G H K S P Q E L K Q D E Q M K R - - - - - - - - - - - - - - PiCdc73
37  - - - - - - I K K M F A R E K R T R G L K Y M M H G - - - - - - - - - - - - - - EhCdc73
151 - - - - - K I H D D F Q K R A S E K I G E Q V E Y D - - - - - - - - - - - - - - TvCdc73
278 E V N S P E V A A A I E A D R D A T R R I T A L E I P V G D S A S I L R A G A G TpCdc73

    - - - - - - - - - - - - - - - - - - - - - - - - - - - - - - - - - - - . . . . . Consensus #1
    - - - - - - - - - - - - - - - - - - - - - - - - - - - - - - - - - - - - - X X P Majority
                      690                 700                 710                 720
140 - - - - - - - - - - - - - - - - - - - - - - - - - - - - - - - - - - - - - - - - TbCdc73
184 - - - - - - - - - - - - - - - - - - - - - - - - - - - - - - - - - - - - - A K P ScCdc73
165 - - - - - - - - - - - - - - - - - - - - - - - - - - - - - - - - - - - - - I K P SpCdc73
293 - - - - - - - - - - - - - - - - - - - - - - - - - - Y D Q E R F K G K E E T E G HsCdc73
298 - - - - - - - - - - - - - - - - - - - - - - - - - - E K K S S D K V S V L Q S S DdCdc73
279 - - - - - - - - - - - - - - - - - - - - - - - - - - F G N E D I M N E I Q I D T NgCdc73
680 I Y H T L Q L D N N I K D N N F E L I K S V S V K E V N V K D V H D N L G N N S PfCdc73
183 - - - - - - - - - - - - - - - - - - - - - - - - - - - - - - - - - - - K F N S N TtCdc73
183 - - - - - - - - - - - - - - - - - - - - - - - - - - I T D R E Y V N R T R T T V PiCdc73
57  - - - - - - - - - - - - - - - - - - - - - - - - - - - - - - - - - - - - - - - - EhCdc73
172 - - - - - - - - - - - - - - - - - - - - - - - - - - - - - - - - - - - - I L R P TvCdc73
318 G K G S A E A N S - - - - - - A A A N A S G G G G T T P G S V K K R D F A R V TpCdc73
```

```
    f a f . . f . . . . f f . f f p . p p p . p b . . p . . . . - - - - - . . p p .  Consensus #1
    I D F X L L X K D A V X E L L X S X N X K S X X K X - - - - - - - - - - - S X X  Majority
                            730                 740                 750                 760
140 - - - - - - - - S A G A N N I L R S I G N G I R I N - - - - - - - - - - - P G F  TbCdc73
187 I N F G Y L I K D A E L K L V Q S I K G S L R G S K L P - - - - - - - - - - P G H  ScCdc73
168 I D F I S L R K D - V I D Y I H A N K A T A S - - - - - - - - - - - - - - - A H  SpCdc73
307 F K I D T M G T Y H G M T L K S V T E G A S A R K T Q T P A - - - - - A Q P V P  HsCdc73
312 V S F A N I L T N Y K K F K I E D E E E K K R K R S S S S S - - - - - S S S S L  DdCdc73
293 T G S M M M K S D P V I E P L S S G N N T S A P P S T T N N - - - - - T F S I N  NgCdc73
720 V D F S R I H N Y F K E E L L N K N N I K N V F I N G I K K N I I V D D Q S K M  PfCdc73
188 I N F K H Y I K Q L D E G K M I T Q N N P L E I K S - - - - - - - - - - - T I N  TtCdc73
197 L D A H K T K F E N V V K T L E L V N A E T K E K I E K A S - - - - - K A S A L  PiCdc73
57  N D N L L R L K E E I F E G Y N L V D S K Q K - - - - - - - - - - - - - - - E N K  EhCdc73
176 I D S V L L N E K D F S E M I T H T N S I V N Y Q Q Q N R D - - - - - N Q Y D S  TvCdc73
352 L E I Y Q E V V Q A E E K A K R A S S S G S K R P A P P G G - - - - - P S S S S  TpCdc73

    . f . p p . . . . . p . - - - - - . . . . . b . f f f f f f . p - - - - - - - -  Consensus #1
    X D X T X K K S K P X - - - - - - - - K X - X T P I I L V P X X - - - - - - - -  Majority
                            770                 780                 790                 800
161 M V P E Y H S Q R N R - - - - - - - - K L N F V P L I L I S P S - - - - - - - -  TbCdc73
218 K G A H G R I S K T N G S - - - - S G G P R K D P I I L I P S A - - - - - - - -  ScCdc73
192 A D E Q E R P A K K R - - - - - - N - - - - R D P I I L L S P S - - - - - - - -  SpCdc73
342 R P V S Q A R P P P N - - - - - - Q K K G S R T P I I L I P A A - - - - - - - -  HsCdc73
347 L S S P N V K S S P N G S P S I I P H L K N R T P I I I V P S S - - - - - - - -  DdCdc73
328 I D E T I K K S V P S T S - - - - K Q V K K P L P I I L I P P G - - - - - - - -  NgCdc73
760 D D K K K I K K K N Y K W I D E I H L V Y K K R P I I I I E K N Q I I T N S D N  PfCdc73
217 Y P C L F K D L R D K G - - - - - - - - - - L K P I I V V P T M - - - - - - - -  TtCdc73
232 L A T T I V R K E Q L P L H R L V K E K I L G T P I I V V P A G - - - - - - - -  PiCdc73
83  T I K T Q K T H D D S - - - - - - - - - - - - - Y Y I I M P S V - - - - - - - -  EhCdc73
211 S A S S L I D A N Q S - - - - - - - - R I F K K P I I L V P K T - - - - - - - -  TvCdc73
387 K S V R G A G S G A A A A V G A S V A K I D G N P I I V V P N A - - - - - - - -  TpCdc73

    - - - - - - - - f p p f f p f f p f b . f f - . a p f f f . p . a b . . . f p .  Consensus #1
    - - - - - - - - R S S L L T X X N I K D F L - X E G K X V P P D E K K X N X D X  Majority
                            810                 820                 830                 840
185 - - - - - - - - V S S V L Q V I N I K D F L - E D G V Y V E P S S R F L N P V T  TbCdc73
246 - - - - - - - - A S S I L T V A N I K Q F L - L E S K Y V N P R N L P S V P N G  ScCdc73
214 - - - - - - - - A S S L L T M H N I K K F L - E E G I F V P P A E A A H A A G G  SpCdc73
368 - - - - - - - - T T S L I T M L N A K D L L - Q D L K F V P S D E K K K Q G C Q  HsCdc73
379 - - - - - - - - L T A T I S L Y N V L E F L - Q H S L F R P T L E K K Q E M A S  DdCdc73
356 - - - - - - - - R S S L L T L Y N I K D F L - Q R G A F V S T D E K K K N I D P  NgCdc73
800 K S I I D N T - R K P I I N K N N V K S F L L H N K L L T T N N D D I L Q N D K  PfCdc73
239 - - - - - - - G R T G N I S L F N A K K F L - A E G K Y E D P E N K D S D N S E  TtCdc73
264 - - - - - - - - F S D L F T M L N A K D F L - E D G V Y V S N M Q K K S E G Q R  PiCdc73
102 - - - - - - - - - S A P L N L D N I Q Q F I - K Q T K Y I K S S L C D T V N G -  EhCdc73
235 - - - - - - - - S Q C A L N N T N I K Q F L - V E N I W S E P D E G G E N - - -  TvCdc73
419 - - - - - - - - M T S C I T M V N A G F F L G K E A T F I P R D Q A V K R P D A  TpCdc73
```

```
      - - - - - . . f . . f . b p f p - - - . f - p . . f . f . f f a p f . . f - - - -  Consensus #1
      - - - - - - X I Q X I E K X X X - - - - - - - G X T I T F X V V D N P K X X - - - -  Majority
                        850                 860                 870                 880
216   G - - D M N V E D A P K H I I V K P G S F L D A D K Y R V A Y R E F R V V N - -  TbCdc73
277   - - - - - - - L V N I E K N F E - - - R I - S R P I R F I I V D N T R M F T - - -  ScCdc73
245   G R G P E L I A L S H K S S N - - - S K - F G T M R F I I V E G T E K F - - - -  SpCdc73
399   R E N E T L I Q R R K D Q M Q P G G T A I S V T V P Y R V V D Q P L K L - - - -  HsCdc73
410   - - - Q N I I K P P M I I P D - - - R I T I Q K V T Y E V I D N I K S L - - - -  DdCdc73
387   - - - - T T P K P K E V I I K - - - H A - K T G T Q Y L V L D I T K N L - - - -  NgCdc73
839   - - - - - N K Q N V F H S T S L I Y K M F N K S I K F L F V E I N H I K K - - -  PfCdc73
271   - - - - - R V I Q I E K N I K - - - - - - - G H N F L F E V Y D N V G T F E S K N  TtCdc73
295   - - - - - K Q Q S M M I I H E - - - - - E D G H V Y T F K V V D T V N R F - - - -  PiCdc73
131   - - - - - - V V D I T Y Q G - - - - - - - - - - - V V Y H F I N K P T N D - - - -  EhCdc73
263   - - - H F V I N H P H T V T G - - - - - - - - K I I D F D V V A D P K L I - - - -  TvCdc73
451   G K R G G T I S I T R K L T S - - - R I G G G D I T F D I I D N P T T R - - - -  TpCdc73

      - - - b f . a f a b f f f f f f p p . . f p f b . - - - . f b f p a - - - - - - - f  Consensus #1
      - - - K P E D W D R V V A V F V X G Q A W Q F K - - - - W K W S D - - - - - - - P  Majority
                        890                 900                 910                 920
252   G P K Q V K N W N H V C A C I V D G N E W Q F N - - - R W F P D E V P - S L C V  TbCdc73
304   - - - K P E Y W D R V V A I F T T G H T W Q F N - - - N Y Q W N S - - - - - - - P  ScCdc73
277   - - - K P D Y W D R V V C V F T T G Q A W Q F R - - - D Y K W S E - - - - - - - P  SpCdc73
435   - - - M P Q D W D R V V A V F V Q G P A W Q F K G - W P W L L P D G - - - - S P  HsCdc73
440   - - - K P E D W Y R V V A A F V Q G E A W Q F K - - - D W K W S N - - - - - - - P  DdCdc73
415   - - - H K N D W E R V V A I F T I G Q L W Q F K D S N N W F S T D - - - - - - - P  NgCdc73
871   - - F T Q N D W K C V I A V I L N S K E S - I K Y - - I F K Y Y P Y Q I - - - T  PfCdc73
300   N P S Q K S K W E R V V G V F V S G Q K Y Q F K G - - - W P K E D - - - - - - N I  TtCdc73
322   - - - R D K D W R S V V G V I V S G Q S W Q F K G - - - W K W K F - - - - - - - P  PiCdc73
151   - - - I P K - - D R I V A V F L I D E D W Q F E D - - - L P F K T - - - - - - - P  EhCdc73
289   - - - K I E D W K Y V V A I F I S G F K W E I N - - - D F H A D D D N R Q H D V  TvCdc73
484   - - L Q K E D W N R V V A V I A Q G A S W Q F K G - - - W R Y S E - - - - - - - P  TpCdc73

      . a f f p b f b p f p f p . a p a . f f . . f . p - f p f . f f . f p b . - - b  Consensus #1
      S X L F X X V K G F Y V K Y X G D Q L P P E V Q N - W N V K I L K X S R X - - K  Majority
                        930                 940                 950                 960
288   S R L F Q R V C G F L P Y F E E D K P P K A L Q E - W H V T P L K E I R R V V K  TbCdc73
332   Q E L F Q R C K G Y Y E H F A G D S V P Q H V Q Q - W N V E K V E L D K N - - K  ScCdc73
305   H Q L F H H V K G F L V Q Y V G D P P H P A T H D - W N V E G I F V E R L - - R  SpCdc73
467   V D I F A K I K A F H I K Y D E V R L D P N V Q K - W D V T V L E L S Y H - - K  HsCdc73
468   A D L F A N I R G F Y V K F D D S N L P D V V K S - W D V K V L H I S K S - - K  DdCdc73
446   S I I F S K K K G F T L A Y E G E A L P Q N T Q N - W N V E K L Y I S R L D S K  NgCdc73
903   T T L F Y P F K N F L E I Y N D D I I P H E L L S Q S N M E I I K L N R D - - H  PfCdc73
332   P S I L D Q V K G F Y L K Y Y D I P T P D Q V A N - W N V K I V S V Y R N - - K  TtCdc73
350   L E V F K K V C G V H I Y N Q G S Q L N P E I K Q - W D V K V L M I H P D - - K  PiCdc73
177   E E C T S K C C C F Y V S L N H Q Q L P M K I W L - R H A Q I L H V C L N - - -  EhCdc73
323   S K L F Q R Y L G I Y V C F D N D Q N Y S T V Q N - W K V K I Y Q I S H A - - Q  TvCdc73
513   V D L F S R A F G F Y V G L E G A A V P N E L K G - W N C K I G K V S R D - - R  TpCdc73
```

```
    b b f a . . f f . . f f . . f a . f f . . b . p . . . . . . . . . . .   Consensus #1
    R H L D X X V A X X F W X X L E X F M X X X K X - - - - - - - - - - -   Majority
                      970                 980                 990
327 S H T H I R Q A S A F W E H L Y L F I D T H P L F K L F T V P P D Q P   TbCdc73
369 R F K D V E V V R Y F W H S L E K E I I S R G Y R                       ScCdc73
342 R H T D R E V V S Q L W D K L E R W M E N R W P L W N G R R             SpCdc73
504 R H L D R P V F L R F W E T L D R Y M V K H K S H L R F                 HsCdc73
505 R H L D H T A Q V E F W N A F D D F T N A K K S Y L N H                 DdCdc73
485 R H T D S T V A R K F W S S L G M E                                     NgCdc73
941 R N D D H L A V Q K F W K K I E H F I L Q K R D S N F Y I P K K N N     PfCdc73
369 R H L D F P A Y E Q F W Q E L E N F M I Q P R N K Q K                   TtCdc73
387 R H L D K V A A K E F W R Y L F A F I K H K L Q                         PiCdc73
213 - - - - - - - Y K Q I F F                                               EhCdc73
360 S F L Q S Q V A N K I W Q E I E N N Y R K I Y H K Q                     TvCdc73
550 R G L D N I C I A S F W N G L D E F M A V H K Q G Y S R M               TpCdc73
```

Consensus 'Consensus #1': When 50% (6) match the residue group of the Consensus show the group label of the Consensus, otherwise show '.'. Residue Groupings of Consensus #1 are: a=(DE), b=(HKR), f=(AFILMPVW), p=(CGNQSTY).

Decoration 'Decoration #1': Shade (with black at 50% fill) residues that match the consensus named 'Consensus #1' exactly.

Decoration 'Decoration #2': Shade (with solid black) residues that match the Consensus exactly.
