# Supplementary material for: Depletion of Trypanosome CTR9 Leads to Gene Expression Defects
Source: PLoS One. 2012 Apr 20;7(4):e34256. doi: 10.1371/journal.pone.0034256 (PMC3332058; doi:10.1371/journal.pone.0034256)
Supplement: Figure S3 — Alignment of Leo1 homologues, made using MegAlign (DNAStar). Details as for Figure S1. Organisms are: Tb- T. brucei, At- Arabidopsis thaliana; Dd- Dictyostelium discoideum XP_637916.1; Tp- Thalassiosira pseudonana XP_002289477.1; Hs-Homo sapiens NP_620147.1; Sc- Saccharomyces cerevisiae Leo1; Ng- Naegleria gruberi XP_002677260.1; Tt- Tetrahymena thermophila EAR92336.1; Pi-Phytophthora infestans XP_002903832.1 (PDF) [file pone.0034256.s003.pdf]

Supplementary Figure S3

Alignment of Leo1 homologues, made using MegAlign (DNAStar). Details as for Figure S1. Organisms are: Tb- *T. brucei*, At- *Arabidopsis thaliana*; Dd- *Dictyostelium discoideum* XP_637916.1; Tp- *Thalassiosira pseudonana* XP_002289477.1; Hs- *Homo sapiens* NP_620147.1; Sc- *Saccharomyces cerevisiae* Leo1; Ng- *Naegleria gruberi* XP_002677260.1; Tt- *Tetrahymena thermophila* EAR92336.1; Pi- *Phytophthora infestans* XP_002903832.1

```
    - - - - - - - - - - - - - - - - - - - - - - - - - - - - - - - - - - - - - - - -  Consensus #1
    - - - - - - - - - - - - - - - - - - - - - - - - - - - - - - - - - - - - - - - -  Majority
                      10                  20                  30                  40
1   - - - - - - - - - - - - - - - - - - - - - - - - - - - - - - - - - - - - - - - -  TbLeo1
1   - - - - - - - - - - - - - - - - - - - - - - - - - - - - - - - - - - - - - - - -  AtLeo1
1   - - - - - - - - - - - - - - - - - - - - - - - - - - - - - - - - - - - - - - - -  DdLeo1
1   - - - - - - - - - - - - - - - - - - - - - - - - - - - - - - - - - - - - - - - -  TpLeo1
1   M A D M E D L F G S D A D S E A E R K D S D S G S D S D S D Q E N A A S G S N A  HsLeo1
1   - - - - - - - - - - - - - - - - - - - - - - - - - - - - - - - - - - - - - - - -  ScLeo1
1   - - - - - - - - - - - - - - - - - - - - - - - - - - - - - - - - - - - - - - - -  NgLeo1
1   - - - - - - - - - - - - - - - - - - - - - - - - - - - - - - - - - - - - - - - -  TtLeo1
1   - - - - - - - - - - - - - - - - - - - - - - - - - - - - - - - - - - - - - - - -  PiLeo1

    - - - - - - - - - - - - - - - - - - - - - f p . a p a . a . - - - - - - . - - - -  Consensus #1
    - - - - - - - - - - - - - - - - - - - - - - - - X X E - X - - - - - - - X - - - -  Majority
                      50                  60                  70                  80
1   - - - - - - - - - - - - - - - - - - M Y R H G L E S E E Q R H E D L L L L P T Q  TbLeo1
1   - - - - - - - - - - - - - - - - - - - - M V K G E K R S E M M L N L F G D N S E  AtLeo1
1   - - - - - - - - - - - - - - - - - - - - - M N D F G E E D E R Q I L E G T F V D  DdLeo1
1   - - - - - - - - - - - - - M A E S N T N A A N L F G E S D S S D E S V G A P P A  TpLeo1
41  S G S E S D Q D E R G D S G Q P S N K E L F G D D S E D E G A S H H S G S D N H  HsLeo1
1   - - - - - - - - - - - - - - - - - - - - - M S S E S P Q D Q - - - - - - - - - -  ScLeo1
1   - - - - - - - - - - - - - - - - - - - - - - - - - - - - - - - - - - - - - - - -  NgLeo1
1   - - - - - - - - - - - - - - - - - - - - - - - - - - - - - - - - - - - - - - - -  TtLeo1
1   - - - - - - - - - - - - - - - - - - - - - - - - - - - - - - - - - - - - - - - -  PiLeo1

    . . . . . p . p . . p f p p p p p p p a a a . - - - - - - - - - - - - - . p . a . p  Consensus #1
    - - - - - X X X E X X A X S N X S S X X E E E - - - - - - - - - - - - - - X D X G  Majority
                      90                 100                 110                 120
23  S D T Q T T E E R T T A N P T T Q G A T D E G - - - - - - - - - - - G S G A A D  TbLeo1
21  E E E I E S E H E C N R R Q P N Y A S D E A E G G V E P - - - - - - E G E G E A  AtLeo1
20  K S S K Q T T N D N E N N N N N N R E E E N - - - - - - - - - - - - E N Q D K N  DdLeo1
28  P A A A E N V E A N R T K I A D S D D N D D G - - - - - - - - - - - A P P P S G  TpLeo1
81  S E R S D N R S E A S E R S D H E D N D P S D V D Q H S G S E A P N D D E D E G  HsLeo1
10  P Q K E Q I S N N V G V T T N S T S N E E T S - - - - - - - - - - - R S Q D D N  ScLeo1
1   - - - - - M T D K A S M E E I F G S D F E E E - - - - - - - - - - - - - - - - -  NgLeo1
1   - - - - - - - - - - - - - - - - - - - - - - - - - - - - - - - - - - - - - - - -  TtLeo1
1   - - - - - M A S S P E A Q S G S S S S S E D E - - - - - - - - - - - - - F D L G  PiLeo1

    . . p f p p p . a p . f a p p a p a f a f a a f f p a - - - - - - - - - a p . . .  Consensus #1
    G X S V X - - S E X X A X X X D K D X X L D D L F X D - - - - - - - - - D X D X X  Majority
                     130                 140                 150                 160
52  G E G A A T Y L T P R A V S V D S D L T L E D L F G P - - - - F F Y V D K S L V  TbLeo1
55  E V E V H G E A E A E S D G E Q G D V E L D P G E S E G E R - - - - - E Q S S Q  AtLeo1
49  V T T K N - D E D N N N D A N D G D D D D D D L F G D K D L - - - - - E E N D N  DdLeo1
57  G V S V Q T N K E G R A D D A D K P A D E P A A A K D S D E - - - - - K V K N P  TpLeo1
121 H R S D G G S H H S E A E G S E K A H S D D E K W G R E D K S D Q S D D E K I Q  HsLeo1
39  V K E V N G N D D T K E E E Q E E D A E L D D L F G D - - - - - - - - D N D D D  ScLeo1
19  - - - - - - - S E - - - E E Q Q Q Q Q Q N E E I Q S S - - - - - - - - - S D N E  NgLeo1
1   - - - - - - - - - - - - - - - M M D E E Q D V L F S E - - - - - - - - - - - - -  TtLeo1
23  G S S L K - - S S G D A C S S S K P V D P D K L F S D - - - - - - - - D S D E E  PiLeo1
```

```
     f . a . p a b p . p b . p a - - . . p a a a a a . a a f a . a a b p - - - - - -  Consensus #1
     V X D D X E R S E X X K S D - - X D X D X D E E X E D X X X E X R Q - - - - - -  Majority
                       170                 180                 190                 200
88   V V D P A L L G S R S L L R R F F G D T I C E T D V D I T S D N E E - - - - - -  TbLeo1
90   E A D P Q E E S E A R D S D - - S D N K E E E H G G R V A K K R R Q E V V E S G  AtLeo1
83   V N S D N E K N K S K K D K - - E D Q D Q E E Q Q E D Q E D E E Y S - - - - - -  DdLeo1
92   P S S S S S A S N A N K N S N L F G D D D S S D D D E F D G N D D G I V G R S G  TpLeo1
161  N S D D E E R A Q G S D E D K L Q N S D D D E K M Q N T D D E E R P Q L S D D E  HsLeo1
71   D D D D V K K S E I E K S D - - S D S D E D D E G E N I N H R S R H - - - - - -  ScLeo1
40   V E E F T H R R V I R S S E - - - - H Q K I A E K E R L E E E Q R K - - - - - -  NgLeo1
13   - - - - - - - - - - - - - - - - - - N E F Q V S K E Q F T F N K N Q - - - - - -  TtLeo1
53   E D D N K E R V E K K G A L S P K K E E K D K E M E D L F G S D Y D - - - - - -  PiLeo1

     - - - . . . a a . f f p p - - - - - - - - - - - - - - - - - - - - - - - - f p a  Consensus #1
     - - - S X E X E X E A S X - - - - - - - - - - - - - - - - - - - - - - - - - - E  Majority
                       210                 220                 230                 240
122  - - - A L R K E V E I A R - - - - - - - - - - - - - - - - - - - - - - - - - - -  TbLeo1
128  S E R S G E K H Y E S E D E E - - - - - - - - - - - - - V D Q T R S P R S P S E  AtLeo1
115  N K K K K K K S K K N K N - - - - - - - - - - - - - - - - - - - - - - R H S T E  DdLeo1
132  G A N S A V N I A A A S G - - - - - - - - - - - - - - - - - K T V A K K P M I M  TpLeo1
201  R Q Q L S E E E K A N S D D E R P V A S D N D D E K Q N S D D E E Q P Q L S D E  HsLeo1
103  R E S L G L D D D E A E E - - - - - - - - - - - - - - - - - - - - - - Q A M Y T  ScLeo1
70   - - - - - L E D Q M L T S - - - - - - - - - - - - - - - - - - - - - - - - - - -  NgLeo1
29   - - - - - K G W Y L K H Q - - - - - - - - - - - - - - - - - - - - - - - - - - -  TtLeo1
87   - - - S E E E E F K A S G - - - - - - - - - - - - - - - - - - - - - - - - V K E  PiLeo1

     . . . . . . a . p p . a . p . p . f p . p a . a a p a a . f . - - - - - - - - -  Consensus #1
     X X - - - - D X - X D X D F D X X X G X X D K E E X E E X I R - - - - - - - - -  Majority
                       250                 260                 270                 280
132  - - - - - - - - - - K E A C V N Y V Y E L F G E E G A A I I G - - - - - - - - -  TbLeo1
155  E K E E V Q V A Q S D V N I R N V F G S S D D E D A E E Y V R N D V E Q D E H R  AtLeo1
133  E D E T M E D A N E N L D F A T G A G E E E E E E E P E L I R - - - - - - - - -  DdLeo1
155  N Q T L G L D S D S D D D K G V G S G A E T K E G Q E E D D D - D - - - - - - -  TpLeo1
241  E K M Q N S D D E R P Q A S D E E H R H S D D E E E Q D H K S E S A R G S D S E  HsLeo1
121  R K F Y G E D A N N F S D Q D E - T T H T F K E E N V E L V R - - - - - - - - -  ScLeo1
78   - - - - - - - - - Q A E H Y D S L L Y K Y D S D G D D E S K Q - - - - - - - - -  NgLeo1
37   - - - - - - - - - - - - - - - Q A V G F C A K E Y N E E H I K - - - - - - - - -  TtLeo1
100  S P A R D G G R S N E D L F G D E A G D R S A S G G G D G D N - - - - - - - - -  PiLeo1

     - - - - - - - - - - - - . . f . f . a f . f p p f f f b . . - - - - - - - - - -  Consensus #1
     - - - - - - - - - - - - - - X - P S X X E X S X V A P K D - - - - - - - - - - -  Majority
                       290                 300                 310                 320
153  - - - - - - - - - T Q E P K P F D C L V S S V P D K Q N V G - - - - - - - - - -  TbLeo1
195  S P I E D E E G S E K D L R P D D M V L D D I I P E E D P - - - - - - - - - - -  AtLeo1
164  - - - - - - - - - - D P I T L T H I E T S N V L P A Q - - - - - - - - - - - - -  DdLeo1
187  - - - - - - - - K P V Y A P P R K M E L L N I A T T D G - - - - - - - - - - - -  TpLeo1
281  D E V L R M K R K N A I A S D S E A D S D T E V P K D N S G T M D L F G G A D D  HsLeo1
151  - - - - - - - - - - H - I I P S K A N V N E T A S H N - - - - - - - - - - - - -  ScLeo1
100  - - - - - - - - - - - - - - V L E V K H S E R P T K D S - - - - - - - - - - - -  NgLeo1
53   - - - - - - - - - - - - - - - - - - - - - - - - - - - - - - - - - - - - - - - -  TtLeo1
131  - - - - - - - - - - G Y S D N M W I P K T P K A P K T - - - - - - - - - - - - -  PiLeo1
```

```
    - - - - - - - - - - - - - - - - - - - - - - - - - - - - - - - - - - - - - - - - Consensus #1
    - - - - - - - - - - - - - - - - - - - - - - - - - - - - - - - - - - - - - - - - Majority
                      330                           340                           350                           360
174 - - - - - - - - - - - - - - - - - - - - - - - - - - - - - - - - - - - - - - - - TbLeo1
224 - - - - - - - - - - - - - - - - - Q Y E S E A E H V E A R Y R E R P V G P P L E V E AtLeo1
181 - - - - - - - - - - - - - - - - - - - - - - - - - - - - - - - - - - - - - - - - DdLeo1
207 - - - - - - - - - - - - - - - - - - - - - - - - - - - - - - - - - - - - - - - - TpLeo1
321 I S S G S D G E D K P P T P G Q P V D E N G L P Q D Q Q E E E P I P E T R I E V HsLeo1
167 - - - - - - - - - - - - - - - - - - - - - - - - - - - - - - - - - - - - - - - - ScLeo1
114 - - - - - - - - - - - - - - - - - - - - - - - - - - - - - - - - - - - - - - - - NgLeo1
53  - - - - - - - - - - - - - - - - - - - - - - - - - - - - - - - - - - - - - - - - TtLeo1
148 - - - - - - - - - - - - - - - - - - - - - - - - - - - - - - - - - - - - - - - - PiLeo1

    - - - - - - - - - - - . . f f f f b f f p f f p - f a f b f f a f . p f . f a a a Consensus #1
    - - - - - - - - - - - - K I F X X K L P N I L G - I D P K P F D P E T F E X E X X Majority
                      370                           380                           390                           400
174 - - - - - - - - L Y E E M W L T D M P K I A P N R Q T L H M E P R P Y V S S N P TbLeo1
249 V P F R P P P G D P V K M N M I K V S N I M G - I D P K P F D A K T F V E E D T AtLeo1
181 - - - - - - - - - - - K I M K L K L L N I L G - I Q P K P F D P I T F E D E E - DdLeo1
207 - - - - - - - N H Q T K I H I T K L P N L V G - I N T A A Y N P E T H N L E D E TpLeo1
361 E I P K V N T D L G N D L Y F V K L P N F L S - V E P R P F D P Q Y Y E D E F E HsLeo1
167 - - - - - - - - - - - E I F Y A R I P N F L T - I D P I P F D P P S F E A K V N ScLeo1
114 - - - - - - - - - - L D L F F L R T P I Q F N - T D E K E F T E E S S L P K N D NgLeo1
53  - - - - - - - - - - - - - - - - Y L E N E Y N - I K S - Q H S K Q L F G I Q E - TtLeo1
148 - - - - - - - - - - A K Y F I S K M P N I L R - L V P E P Y T K E A I R A E M D PiLeo1

    - - . f . p a a . p - - - - - - - - b . . . a p . f b f b f . b a . p p b f f b a Consensus #1
    - - - M X S X E X S - - - - - - - - R Y K X E N T I R W R X X R D E X G X P X K E Majority
                      410                           420                           430                           440
206 S A P L R S T E R S - - - - - - - L Y T T Q N V I R W S I G - - - G R D K L F V TbLeo1
288 - - F M T D E P G A K N - - - - R I R L D N N I V R H R F V K S R D G K T Y S E AtLeo1
208 - - A M N G D E K S - - - - - - - K F N V E S V I R W R W G L D L N G R P A K E DdLeo1
239 E E Y Y R G Y V H N M I R - - - W R Y K N K P E D E T E I V R D E N G H P I R E TpLeo1
400 D E E M L D E E G R T R - - - - L K L K V E N T I R W R I R R D E E G N E I K E HsLeo1
195 - - E R A S N S A S R E D Q L D D R L I D E N T V R W R Y S R D K D Q H V F K E ScLeo1
143 - - - - - - - - - - - - - - - - - - - - - L Y T I R W K K - N L E I G - - E I E NgLeo1
74  - - - - - - - - - - - - - - - - - - - - - S S T I R Y R K - - N N E G N - - L E TtLeo1
177 - - - N P S D E T L Y R N Y V R W R Y K R D P A T G R V L L D D K T K L P L R E PiLeo1

    p p p b f f . f p a p p p p f . f p p a f f a f p . p a - - - - - - - - - - - - - Consensus #1
    S N X R I V Q W S D G S Y S L X V G N E V L X X L E Q D - - - - - - - - - - - - Majority
                      450                           460                           470                           480
236 S N A R I V R W S D G S T T L R V G S D S F P L Q L S R D N A I T M L A S P S A TbLeo1
322 S N A R F V R W S D G S L Q L L I G N E V L N I I E Q D - - - - - - - - - - - - AtLeo1
239 S N T R L V T W S D G S S H L Y I G N E V L E I K E Q P - - - - - - - - - - - - DdLeo1
276 S N T R L V K W S D G S Y T L H V G Q E V L E V D N L D S S V P L D E K A E G A TpLeo1
436 S N A R I V K W S D G S M S L H L G N E V F D V Y K A P - - - - - - - - - - - - HsLeo1
233 S N T Q I V Q W S D G T Y S L K V G E E C T D I L V N D - - - - - - - - - - - - ScLeo1
159 S N T R V V Q Y E D G S Y Q V F V G N E V I L N L E A K - - - - - - - - - - - - NgLeo1
89  S N A K I I E W D D G S Y H L M V G D Q F F K L N L K D - - - - - - - - - - - - TtLeo1
214 S N S K L V Q W E D G T F S M F V G K E A L T L S R Q K L A N S F L F V N - - - PiLeo1
```

```
    - - - - - - - - - - - - - f p p a p . b f p f b p p p - f - - - - - - - - - - -  Consensus #1
    - - - - - - - - - - - - - X Q N D X X H L X X S Q X X - - - - - - - - - - - - -  Majority
                        490                 500                 510                 520
276 I S K G D L T L P A M V S V I N P S K H F V A E C N S A A S I E K A V N E E N Q  TbLeo1
350 - - - - - - - - - - - - - A K E D Q N H L F I K H E K G I - - - - - - - - - - -  AtLeo1
267 - - - - - - - - - - - - - I Q N E Q F Y V Y S S Q D G - F - - - - - - - - - - -  DdLeo1
316 G G G N - - - - - A L A G F A G I N G Y L Y Y S Q K A R I R P P S K R E L E N A  TpLeo1
464 - - - - - - - - - - - - - I Q G D H N H L F I R Q G I - - - - - - - - - - - - -  HsLeo1
261 - - - - - - - - - - - - - T S N T F L T V S H D Q Q E - I - - - - - - - - - - -  ScLeo1
187 - - - - - - - - - K - - Y T N Y N N L H L F E K V S P - - - - - - - - - - - - -  NgLeo1
117 - - - - - - - - - - - - - F Q A E G C S L Y E R P N N - - - - - - - - - - - - -  TtLeo1
251 - - - - - - - - - - - - E M A S D S P H F Q D S D D V V P - - - G Q E S - - - -  PiLeo1

    - - - - - - - - - - - - - - - - - - - - - - - - - - - - - - f p p p p - - . - -  Consensus #1
    - - - - - - - - - - - - - - - - - - - - - - - - - - - - - - L E C X G - - X - -  Majority
                        530                 540                 550                 560
316 R I R S V S A Q H N - - - - - L A Y S V P T L P S V D W N N L A A S G T P E G E  TbLeo1
366 - - - - - - - - - - - - - - - - - - - - - - - - - - - - - - L Q S Q G - - R - -  AtLeo1
282 - - - - - - - - - - - - - - - - - - - - - - - - - - - - - - I E C E G - - K - -  DdLeo1
351 N K E G V E G K E K D D I M D D S S D E D A P A Q P A G T V L E C L G - - P - -  TpLeo1
478 - - - - - - - - - - - - - - - - - - - - - - - - - - - - - - - G L Q G Q A V - -  HsLeo1
276 - - - - - - - - - - - - - - - - - - - - - - - - - - - - - - I Q C Y E G G E - -  ScLeo1
203 - - - - - - - - - - - - - - - - - - - - - - - - - - - - - - F C Y Y G H G I - -  NgLeo1
131 - - - - - - - - - - - - - - - - - - - - - - - - - - - - - - - - - - - - - - - -  TtLeo1
272 - - - - - - - - - - - - - - - - - - - - - - - - - - - - - V L E C H A - - R - -  PiLeo1

    - f . b b f p f b f p p f p p b . b b p f p f . f f p b p p b p f b - f b p f f  Consensus #1
    - I K X X L X F R P X S T X S K X H R K L T X X V X G R X Q K X S K - I K X X X  Majority
                        570                 580                 590                 600
351 F L A R S Y N L R K K M M E Q R K N R G E P M T L T E Q L Q M E S E L F E K L Q  TbLeo1
372 - I L K K M R F T P S S L T S N S H R L L T A I V E S R Q K K A F K - V K N C V  AtLeo1
288 - I D S R L S I R P T N I K S K V H Q R L S E N V A K R T V K V S K - I K S I H  DdLeo1
387 - I V S R F A P R P S S L A S E A H R N L T I A V R Q R N V K R A R - I A E I V  TpLeo1
485 - F K T K L I F R P H S T D S A T H R K M T L S L A D R C S K T Q K I R I L P M  HsLeo1
284 - I K K T L M F I P T S T N S K I H Q K L S K A V I R R N Q R Q S K G P G T Y I  ScLeo1
211 - F T H R L N S K A T S T Q T K G R D I A L K L T K G T I I T K I K - - K I V L  NgLeo1
131 - - - - - - I N Q Q S F L T D Q I I P Q I F V D K I G T I Q K N V K              TtLeo1
279 - L K E K F T I R P M T T A S K S H R S L T M S M R A K H N K S V Q K L K E Y I  PiLeo1

    p p f a f a b a b . . b . b . . . a f f b . p p b b p p p p . b . b . p p - - -  Consensus #1
    T X L D P E X E K E K R E K E E X Q A L R X X T R X K Q Q X X R X X R X X - - -  Majority
                        610                 620                 630                 640
391 S L S A K E L L E Q Q Q E Q E R Q A A L R S L T R Q N Q I A H R R R F E R T V E  TbLeo1
410 T D I D P E R E K E K R E K A E S Q N L K A S T K L S Q A R E K I K R K Y P L P  AtLeo1
326 T I L D P E K E K E K R E K E L L D A L R E K K K Q K D D S Y R P N R G R - - -  DdLeo1
425 T D F D P E K E K L A R I K G K D D I A K S Q A R S N S G G R R S G G S S - - -  TpLeo1
524 A G R D P E C Q R T E M I K K E E F R L R A S I R R E S Q Q R R M R E K Q - - -  HsLeo1
323 V S M D P E V E K K E L E R K Q S Q I L R D R R R R Q L K - - E K E K Q E - - -  ScLeo1
248 T S K R P E T E E T K K V M E E A Q S L K K K Q Q K K Q Q - - - - K Y Q N - - -  NgLeo1
158                                                                                  TtLeo1
318 S E L D G E R E Q E Q R V K I T D E K L R L Q N R K K A R Q G Y E Y D R E - - -  PiLeo1
```

```
    - . p . p f . p p p f a a p p a p a - - - p a a p b . p . . . . a . f . f a . a Consensus #1
    - X K R G L X X G Y L E D X X D D D - - - Y X D X R X N - - X X E D X E X D K X Majority
                        650                           660                           670                           680

431 L E G G E Y R E G H N S M G G G N T G L N G G D F D N N D - E D E E I E D D Y E TbLeo1
450 V E R R Q L S T G Y L E D A L D E D - - - D E D Y R S N R G Y E E D L E A E A Q AtLeo1
363 G G K S G S L I A S Y L E N D D D D - - - Y D S Y R N E - - K G D D F V V D D D DdLeo1
462 - R K R G M N A G Y L E D E D D Y D G V N L G K L K R S T M R G N N Y D S D E E TpLeo1
561 - H Q R G L S A S Y L E P D R Y D E - - - E E E G E E S - - - - I S I A A I K N HsLeo1
358 S P D A A F E T G F R K Q N S P T T - - - Y G A S R R N E Y E E D D F L V D D D ScLeo1
281 - - T L T L D N A S L E D A G S S S - - - D E E G N V R K I K R N T L R K P K R NgLeo1
158                                                                                 TtLeo1
355 - R S S R M D A Q F L E E G Y D A I D - - Y D D D E H V G A I K E Q E G K R K S PiLeo1

    - - - - - - - - - - - - - - - - - - - - - - - - - - - - - - - - - - - - . . a . Consensus #1
    - - - - - - - - - - - - - - - - - - - - - - - - - - - - - - - - - - - - X - E E Majority
                        690                           700                           710                           720

470 T - - - - - - - - - - - - - - - - - - - - - - - - - - - - - - - - - - - M L E N TbLeo1
487 R E R R I L N A K K S H K G I P G R S S M T S A R P S R R Q M E Y S E S E R E E AtLeo1
398 - - - - - - - - - - - - - - - - - - - - - - - - - - - - - - - - - - - - E D E E DdLeo1
501 - - - - - - - - - - - - - - - - - - - - - - - - - - - - - - - - - - - - - M D Y TpLeo1
593 - - - - - - - - - - - - - - - - - - - - - - - - - - - - - - - - - - - - - - R Y HsLeo1
395 - - - - - - - - - - - - - - - - - - - - - - - - - - - - - - - - - - - - E E E E ScLeo1
316 - - - - - - - - - - - - - - - - - - - - - - - - - - - - - - - - - - - - - - - - NgLeo1
158                                                                                 TtLeo1
392 - - - - - - - - - - - - - - - - - - - - - - - - - - - - - - - - - S - - A V H R PiLeo1

    . . p . b a a a a a - - - - - - - - - p - . . p a a p a p p p a . a . f . b . b . Consensus #1
    X E X Y R E E E D X - - - - - - - - - X - - E S D E A E E G S D E D X A X R L X X Majority
                        730                           740                           750                           760

475 V R R K R Q R E D D E A D A K H A R N I R L Y E Q A R A G Q Y G S L I D S M Q D TbLeo1
527 S E Y E T E E E E E E K S P A R G R G K D S E D E Y E E D A E E D E E E R G K S AtLeo1
402 D D D Y K E S D D D G S D S S D D D G - S E S G E S E S D S D G D N K S K K K E DdLeo1
504 G E D S D E E E D E W T K N - - - K A R K P S A R A S G G V D N N V P T K N Q T TpLeo1
595 K G G I R E E R A R - - - - - - - - - - I Y S S D S D E G S E E D K A Q R L L K HsLeo1
399 A A F D D E E D D N - - - - - - - - - - - E E E E E E E D A D E E N A S R L R - ScLeo1
316 S - S Y E D E E E S - - - - - - - - - - - - A D S A S T G S D E E S A D S Y S - NgLeo1
158                                                                                 TtLeo1
397 K H G V R R P V P G - - - - - - - - G - - G F D E Y R S S Q R Q R E L E R E R E PiLeo1

    . . - . - - - - - - - - - - - - - - - - - - - . p p p . . . . . f . . a a a a a a Consensus #1
    X X - - - - - - - - - - - - - - - - - - - - - X X S X E G A G V X S D E E E E E Majority
                        770                           780                           790                           800

515 L L S Q L P D G S I V R E S V D E T V E F L R A G S F S P A V V I K E V P L M L TbLeo1
567 N R Y S D E D E E E E E V A G G R A E K D H R G S G R K R K G I E S D E E E S P AtLeo1
441 N K N S N - - - - - - - - - - - - - - - D N K N N K S R K Q D F D D D E D E D E DdLeo1
541 P D F G D - - - - - - - - - - - - - - - - - - S S S E E G E V V F G D D E D E D TpLeo1
625 A K - - - - - - - - - - - - - - - - - - - - - K L I S D E E G E P S G K R K A E HsLeo1
427 - - - - - - - - - - - - - - - - - - - - - - - N L K R E G A A M Y R E E E E E E ScLeo1
342 - - - - - - - - - - - - - - - - - - - - - - - - - - - - - - - - - - - - - S D D NgLeo1
158                                                                                 TtLeo1
427 G E - - - - - - - - - - - - - - - - - - - - R S D S D N D A G V G E G E D D E E PiLeo1
```

```
    a . f f f p b b . - - - - - b b . f f a a a a a a . . .    Consensus #1
    D D R A S T K R - - - - - - K X A V I D D D D X X - - -    Majority

                   810                 820

555 E G V A S E C P D V D I G K V R E E L R K L H E R K        TbLeo1
607 P R K A P T H R - - - - - - R K A V I D D S D E D          AtLeo1
466 D L I V S T K K V - - - - - - K R R V I D D D D D D E D    DdLeo1
563 D A A M F K K R S G G E A K K K A V L D D D D D            TpLeo1
644 D D D K A N K K H - - - - - - K K Y V I S D E E E E D D D  HsLeo1
444 K D R S E T K R R - - - - - - R V A V I E D D E D E D      ScLeo1
345 S S R A S K K R                                            NgLeo1
158                                                            TtLeo1
447 E E M V I R S H K - - - - - - K R R T V D E E D S E        PiLeo1
```

Consensus 'Consensus #1': When 44% (4) match the residue group of the Consensus show the group label of the Consensus, otherwise show '.'. Residue Groupings of Consensus #1 are: a=(DE), b=(HKR), f=(AFILMPVW), p=(CGNQSTY).

Decoration 'Decoration #1': Shade (with black at 50% fill) residues that match the consensus named 'Consensus #1' exactly.

Decoration 'Decoration #2': Shade (with solid black) residues that match the Consensus exactly.
