# Supplementary material for: Depletion of Trypanosome CTR9 Leads to Gene Expression Defects
Source: PLoS One. 2012 Apr 20;7(4):e34256. doi: 10.1371/journal.pone.0034256 (PMC3332058; doi:10.1371/journal.pone.0034256)

Supplementary Figure S4 _ TbCTR9-RPB1 co-immunoprecipitation

Method: Bloodstream-form trypanosomes cell lines expressing TAP-tagged TbCTR9 were used (lanes 5-8), with cells expressing TAP-tag alone as control (lanes 1-4). Protein expression was induced for 24hrs. 1.5 x 10^8 cells were collected by centrifugation and washed once with 1x PBS. Cells were lysed in 500µl breakage buffer as previously described [42, 45]. The concentration of NaCl was then adjusted to 150mM and cell debris was removed by centrifugation (4◦C) at 13000 rpm in the microfuge for 30 minutes. IgG beads were washed as previously described then clarified cell lysates was added prior to incubation for 2 hrs at 4°C in a microfuge tube rotating at 15 rpm. The unbound fraction was collected by centrifugation. The beads were washed 4X with IPP150 buffer [42, 45] and the protein recovered by boiling the beads in 1X Laemmli lysis buffer.
Proteins were separated on an 8% SDS-PAGE gel and transferred overnight to nitrocellulose membranes at 30V. Protein detection by ECL kit (GE Healthcare) was done using (1:1000) anti-RPB1 (unpublished, a kind gift from Günzl laboratory); (1:2000) anti- PAP (peroxidase– antiperoxidase complex; for Protein A detection (Sigma)) for TAP. The loading was equivalent to 5x10^6 cells for lysate (lanes 1,8) and unbound (Lanes 2,7) fractions; For wash 4 (TCA-concentrated, lanes 3 and 6) and the bead-bound fraction (lanes 4 and 5), the whole preparation was loaded.

Results: A good signal of the bound CTR9-TAP protein was detected (lane 5) but there was no detectable RBP1 signal (lane 5). Similar results were obtained by immunoprecipiation of CTR9-myc (not shown).

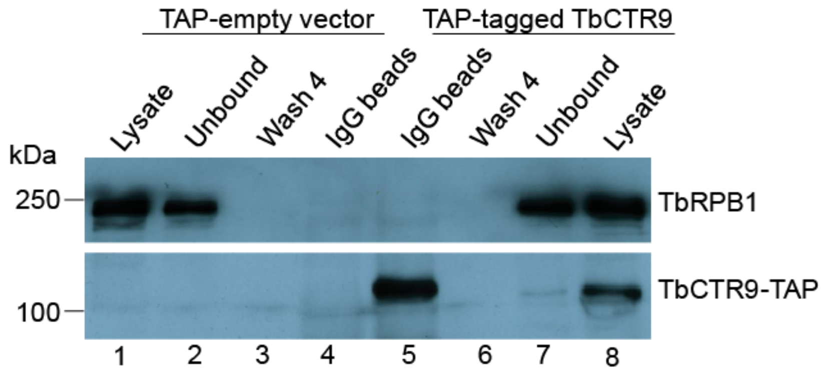

Supplement: Figure S4 — TbCTR9-RPB1 co-immunoprecipitation. Method: Bloodstream-form trypanosomes cell lines expressing TAP-tagged TbCTR9 were used (lanes 5–8), with cells expressing TAP-tag alone as control (lanes 1–4). Protein expression was induced for 24 hrs. 1.5×10^8^ cells were collected by centrifugation and washed once with 1×PBS. Cells were lysed in 500 µl breakage buffer as previously described [42], [45]. The concentration of NaCl was then adjusted to 150 mM and cell debris was removed by centrifugation (4°C) at 13000 rpm in the microfuge for 30 minutes. IgG beads were washed as previously described then clarified cell lysates was added prior to incubation for 2 hrs at 4°C in a microfuge tube rotating at 15 rpm. The unbound fraction was collected by centrifugation. The beads were washed 4× with IPP150 buffer [42], [45] and the protein recovered by boiling the beads in 1× Laemmli lysis buffer. Proteins were separated on an 8% SDS-PAGE gel and transferred overnight to nitrocellulose membranes at 30 V. Protein detection by ECL kit (GE Healthcare) was done using (1∶1000) anti-RPB1 (unpublished, a kind gift from Günzl laboratory); (1∶2000) anti- PAP (peroxidase– antiperoxidase complex; for Protein A detection (Sigma)) for TAP. The loading was equivalent to 5×10^6^ cells for lysate (lanes 1,8) and unbound (Lanes 2,7) fractions; For wash 4 (TCA-concentrated, lanes 3 and 6) and the bead-bound fraction (lanes 4 and 5), the whole preparation was loaded. Results: A good signal of the bound CTR9-TAP protein was detected (lane 5) but there was no detectable RBP1 signal (lane 5). Similar results were obtained by immunoprecipiation of CTR9-myc (not shown). (PDF) [file pone.0034256.s004.pdf]
